# Efficacy and safety of anti-angiogenic drug monotherapy and combination therapy for ovarian cancer: a meta-analysis and trial sequential analysis of randomized controlled trials

**DOI:** 10.3389/fphar.2024.1423891

**Published:** 2024-05-27

**Authors:** Yao Xie, Fei Zhou

**Affiliations:** Department of Obstetrics and Gynaecology, Sichuan Provincial People’s Hospital, School of Medicine, University of Electronic Science and Technology of China, Chengdu, China

**Keywords:** anti-angiogenic drugs, VEGF, bevacizumab, ovarian cancer, monotherapy, combination therapy, meta-analysis

## Abstract

**Background:**

As the development of novel anti-angiogenic drugs and the continuous evolution of guideline recommendations, the efficacy and safety of anti-angiogenic agents in ovarian cancer (OC) remains unclear. Consequently, a meta-analysis was carried out to assess the efficacy and safety of anti-angiogenic drug monotherapy and combination therapy for OC.

**Methods:**

An exhaustive literature review was performed across multiple databases, including PubMed, Embase, Web of Science, and Cochrane, encompassing all relevant randomized controlled trials (RCTs) up until 6 April 2024. The evaluation of efficacy outcomes incorporated progression-free survival (PFS), overall survival (OS), and objective response rate (ORR). Safety was assessed through the occurrence of any grade adverse events (AEs) and grade ≥3 AEs. Synthesis of the data involved the calculation of hazard ratios (HRs), relative risks (RRs), and their corresponding 95% confidence intervals (CIs) and prediction intervals (PIs). Trial sequential analysis was executed employing TSA v0.9.5.10 Beta software, STATA 12.0, and R software 4.3.1.

**Results:**

In this meta-analysis, 35 RCTs were included, encompassing 16,199 subjects in total. The overall analysis indicated that anti-angiogenic drug combination therapy significantly improved PFS (HR [95% CI] = 0.678 [0.606–0.759], 95% PI: 0.415–1.108), OS (HR [95% CI] = 0.917 [0.870–0.966], 95% PI: 0.851–0.984), and ORR (RR [95% CI] = 1.441 [1.287–1.614], 95% PI: 1.032–2.014), but also increased the incidence of grade ≥3 AEs (RR [95% CI] = 1.137 [1.099–1.177], 95% PI: 1.011–1.252). The analysis did not corroborate any benefit of anti-angiogenic monotherapy over placebo concerning PFS (HR [95% CI] = 0.956 [0.709–1.288], 95% PI: 0.345–2.645) and OS (HR [95% CI] = 1.039 [0.921–1.173], 95% PI: 0.824–1.331). However, it was observed that monotherapy with anti-angiogenic drugs did increase the incidence of any grade AEs (RR [95% CI] = 1.072 [1.036–1.109], 95% PI: 0.709–1.592).

**Conclusion:**

Our study confirmed the PFS, OS, and ORR benefits of anti-angiogenic drug combination therapy for OC patients. The efficacy results of anti-angiogenic monotherapy necessitates further evaluation as more RCTs become available. Clinicians should be vigilant of AEs when administering anti-angiogenic agents in a clinical setting.

## 1 Introduction

Ovarian cancer (OC) stands as the primary cause of death related to gynecologic cancer and the fifth most prevalent malignancy, thereby posing a substantial global health risk to women (Siegel et al., 2021). The difficulty in early-stage detection of OC often leads to diagnoses at advanced stages, contributing to a reduced 5-year relative survival rate (Wang et al., 2021). The current standard of care for newly diagnosed patients typically encompasses cytoreductive surgery and platinum-based systemic chemotherapy, with the optional inclusion of bevacizumab. Even with optimal treatment leading to complete remission, about 70% of patients experience a recurrence within 5 years (Hope et al., 2010; McGee et al., 2017). Notably, recurrence rates are nearly 25% for those in early stages and exceed 80% in advanced stages (Garzon et al., 2020). Despite the availability of multiple active therapies for recurrent OC, such as targeted therapy (e.g., poly ADP-ribose polymerase [PARP] inhibitors), chemotherapy, and immunotherapy, the median survival post-recurrence remains less than 3 years, underscoring the critical need to explore new therapeutic options for this patient group (Richardson et al., 2018).

New therapeutic agents, particularly those inhibiting angiogenesis, have shown considerable potential for the treatment of OC. Aberrant angiogenesis, a defining characteristic of solid tumors, is instrumental in tumor advancement (Jászai et al., 2019). By interfering with the formation of blood vessels, anti-angiogenic medications impede the nutrient supply to tumor cells, both by causing damage to the established tumor vasculature and by blocking the creation of new blood vessels (Abdalla et al., 2018). Additionally, these treatments may induce normalization of the tumor vasculature, reversing tumor microenvironment hypoxia, reducing the tumor’s aggressive nature, and augmenting the effectiveness of traditional therapies (Teleanu et al., 2019). The efficacy of angiogenesis inhibitors results from intricate interactions among various pathways, including numerous angiogenic factors like angiopoietin, vascular endothelial growth factor (VEGF), and VEGF receptor (VEGFR) (Saman et al., 2020). Recently, a variety of anti-VEGF strategies, including monoclonal antibodies against VEGF (for instance, bevacizumab) and VEGFR inhibitors (such as cediranib, pazopanib, sorafenib, and apatinib), have undergone evaluation in OC patients (Monk et al., 2016a). The AURELIA trial, a phase III randomized trial, revealed that OC patients experienced a notable extension in progression-free survival (PFS) when treated with a regimen of bevacizumab in combination with chemotherapy *versus* chemotherapy alone. The trial also recorded an enhancement in the objective response rate (ORR) by 15.5% over chemotherapy exclusively. Nonetheless, the addition of bevacizumab to chemotherapy did not yield a statistically significant increase in overall survival (OS) (Pujade-Lauraine et al., 2014).

In the comparison of combined therapy involving angiogenesis inhibitors and standard chemotherapy *versus* conventional chemotherapy alone, a number of randomized controlled trials (RCTs) have illustrated an enhancement in PFS. Nevertheless, debates persist regarding the OS advantage and the safety profile of these combined treatments (Chekerov et al., 2018; Ray-Coquard et al., 2020; Pignata et al., 2021). Prior meta-analyses have explored the efficacy and toxicity of anti-angiogenic drugs in different subtypes of OC (Yi et al., 2017; Helali et al., 2022; Zhang et al., 2022). However, there is a lack of a comprehensive meta-analysis to evaluate the effects of monotherapy or combination therapy with anti-angiogenic drugs on OC. Moreover, multiple RCTs have published the latest relevant clinical results in recent years (Liu et al., 2022; Roque et al., 2022; Ferron et al., 2023; Nicum et al., 2024). Therefore, we conducted a meta-analysis to systematically assess the efficacy and safety of anti-angiogenic drug monotherapy or combined with chemotherapy or PARP inhibitors in the treatment of OC.

## 2 Materials and methods

### 2.1 Study design

The methodology and reporting of our study were aligned with the guidelines delineated in the Preferred Reporting Items for Systematic Reviews and Meta-Analyses (PRISMA) (Page et al., 2021). Furthermore, our study protocol was registered within the PROSPERO database (registration number: CRD42024534864). Given the nature of this research as a meta-analysis synthesizing findings from existing literature, it does not necessitate ethical approval and informed consent, as it neither engages with ethics nor patient privacy.

### 2.2 Search strategy

Our comprehensive search encompassed the databases of PubMed, Web of Science, Embase, and the Cochrane Library of clinical trials, aiming to identify all relevant articles published in English until 6 April 2024. The key search terms employed were: (“anti-angiogenic”, “angiogenesis inhibitor”, “antiangiogenetic”, “anti-angiogenesis”, “vascular endothelial growth factor”, “VEGF”, “VEGFR”, “anti-VEGF”) OR (“bevacizumab”, “cediranib”, “pazopanib”, “afibercept”, “nintedanib”, “sorafenib”, “trebananib”, “avastin”, “recentin”, “votrient”, “perifosine”) AND (“ovar*” AND “cancer*”, “tumor*”, “tumour*”, “carcinoma*”, “neoplasm*”, “malignan*”). A thorough description of the search strategy can be found in Supplementary Files S1. We also manually scrutinized references cited in pertinent review articles to uncover additional studies that may meet the eligibility criteria.

### 2.3 Study selection

Eligibility for study selection was determined by the following criteria: 1) RCTs; 2) the participants are adult women (aged 18 and above) diagnosed with OC at any stage through histological examination; 3) intervention: monotherapy with anti-angiogenic medication or its combination with chemotherapy or PARP inhibitors; 4) comparison: treatment with placebo alone or chemotherapy (alone or plus placebo) or PARP inhibitors (alone or plus placebo); 5) outcomes: PFS, OS, ORR, adverse events (AEs) of any grade, or grade ≥3 AEs. Studies were excluded based on the following: 1) retrospective studies and non-interventional, non-comparative or single-arm trials; 2) studies lacking pertinent outcomes or presenting duplicated data; 3) trial design involving both the intervention and control groups receiving anti-angiogenic drugs; 4) literature reviews, case reports, conference abstracts, commentaries, and study protocols.

### 2.4 Data extraction and quality assessment

Two independent reviewers conducted the study screening, selection, exclusion, and extraction of data. From each RCT, we collated details such as the name of the lead author, year of publication, trial name and phase, patient condition, variety of anti-angiogenic medication used, number of participants and their median age, the doses and cycles of drugs used in the anti-angiogenic agent treatment group and the control group, duration of follow-up, and the outcomes in meta-analysis. PFS and OS were designated as the primary endpoints for this meta-analysis, with ORR, AEs of any grade, and grade ≥3 AEs serving as secondary endpoints. When encountering multiple reports from a single trial, preference was given to the most updated or complete report offering the necessary details. If PFS or OS outcomes were not available directly, the Engauge Digitizer Version 10.8 tool (available at http://markummitchell.github.io/engauge-digitizer/) and Tierney et al.’s proposed methodology (Tierney et al., 2007) were employed to derive data from Kaplan-Meier curves (Xie et al., 2022).

The quality of the RCTs was evaluated utilizing the modified Jadad scale (Jadad et al., 1996), which includes criteria such as the process of randomization, concealment of randomization, implementation of double-blinding, and the tracking of withdrawals and dropouts. Trials were categorized based on their quality with scores ranging from 0 to 3 indicating low quality, while scores from 4 to 7 signified high-quality research.

### 2.5 Statistical analysis

Statistical analyses were carried out using R software Version 4.3.1 and STATA Version 12.0. We calculated the combined hazard ratios (HRs) along with their 95% confidence intervals (CIs) for both PFS and OS. Dichotomous data outcomes were synthesized by computing relative risks (RRs) and delineating these with 95% CIs. We employed I^2^ statistics, Cochran’s Q test, and the 95% prediction interval (PI) to assess heterogeneity across studies (Bowden et al., 2011; IntHout et al., 2016). Findings with I^2^ exceeding 50% or a *p*-value less than 0.10 were deemed to show significant heterogeneity, prompting the use of a random-effects model; if not, we used the fixed-effects model (Higgins et al., 2002). We performed subgroup analysis considering OC subtypes or the types of anti-angiogenic agents. To identify potential sources of heterogeneity, we conducted a sensitivity analysis. Furthermore, the trim-and-fill method was employed to detect and adjust for any publication bias (Duval et al., 2000). A two-sided *p* < 0.05 was considered statistically significant.

### 2.6 Trial sequential analysis

We conducted a trial sequential analysis (TSA) to determine whether the compiled data met the required information size (RIS) for a conclusive finding (Wetterslev et al., 2017). This methodological approach, applied to dichotomous outcomes, utilized TSA software v0.9.5.10 Beta (accessible at www.ctu.dk/tsa). The RIS was calculated, and O’Brien-Fleming α-spending boundaries were established, based on a 5% type I error and a 20% type II error, both set for two-side tests. We engaged STATA Version 12.0, employing the *metacumbounds* and *rsource* function, and R software Version 4.3.1, using the *foreign* and *ldbounds* packages, to execute TSA on the PFS and OS data, adopting the *a priori* information size (APIS) approach. The crossing of the cumulative Z-curve over the trial sequential monitoring boundary or the RIS (or APIS) threshold was interpreted as an indication that no additional trials are necessary, and the evidence could be considered conclusive.

## 3 Results

### 3.1 Literature search

**FIGURE 1:**
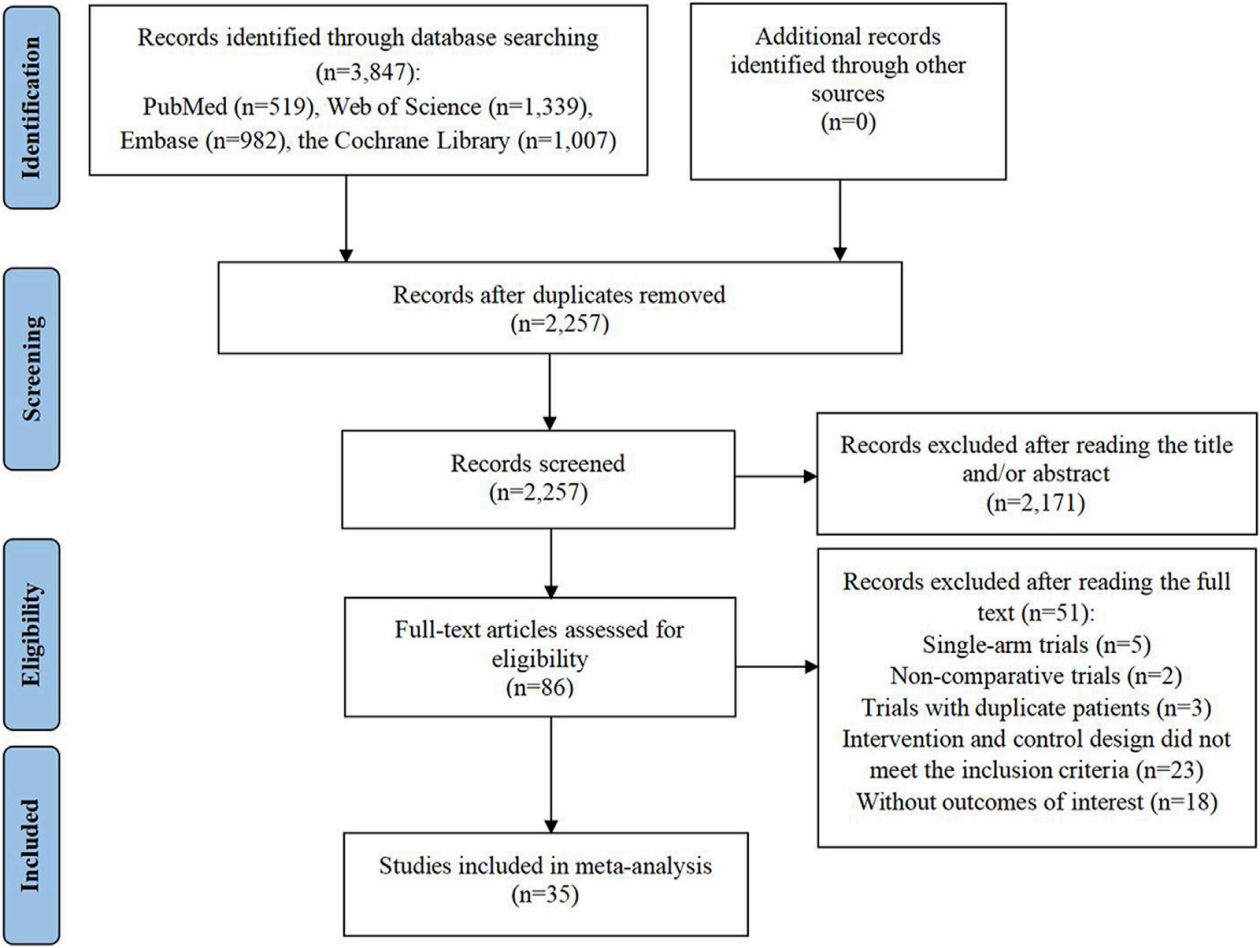
Flow diagram of the process of study selection.

The preliminary search of the database yielded 3,847 entries. Following the removal of 1,590 duplicate entries, a set of 2,257 records persisted for further scrutiny. Out of these, 2,171 were discarded due to irrelevance indicated by their titles or abstracts, leaving 86 articles for full-text review regarding their eligibility. Upon detailed examination, 51 studies were deemed unfit for inclusion: 5 were single-arm clinical trials; 2 were non-comparative clinical studies; 3 trials included duplicate patient data; 23 trials exhibited intervention and control designs that did not align with the inclusion criteria; and 18 articles failed to report the necessary outcome data. Finally, 35 RCTs were selected for inclusion in the meta-analysis (Aghajanian et al., 2012; Aghajanian et al., 2015; Burger et al., 2011; Chekerov et al., 2018; Coleman et al., 2017; du Bois et al., 2014; du Bois et al., 2016; Duska et al., 2020; Ferron et al., 2023; Gore et al., 2019; Gotlieb et al., 2012; Hall et al., 2020; Herzog et al., 2013; Karlan et al., 2012; Kim et al., 2018; Ledermann et al., 2021; Ledermann et al., 2016; Ledermann et al., 2011; Liu et al., 2019; Liu et al., 2022; Marth et al., 2017; Monk et al., 2016b; Nicum et al., 2024; Oza et al., 2015; Pignata et al., 2021; Pignata et al., 2015; Pujade-Lauraine et al., 2014; Ray-Coquard et al., 2020; Richardson et al., 2018; Roque et al., 2022; Shoji et al., 2022; Tewari et al., 2019; Vergote et al., 2019a; Vergote et al., 2019b; Wang et al., 2022) (Figure 1).

### 3.2 Study characteristics and quality assessment

Table 1 provided a detailed overview of the characteristics of the RCTs and the participants that were incorporated into the study. This research encompassed a total of 35 RCTs, which included 15 phase 2 trials and 20 phase 3 trials, all of which were published in English between the years 2011 and 2024. The study population consisted of 8,839 OC patients who were assigned to the anti-angiogenic agent treatment group, while 7,360 patients were administered either a placebo alone or underwent drug therapy that did not involve anti-angiogenic agents. The anti-angiogenic drugs utilized were categorized into VEGF inhibitors (specifically bevacizumab), VEGFR inhibitors (which included pazopanib, cediranib, apatinib, sorafenib, and nintedanib), and angiopoietin inhibitors (solely trebananib). The design of anti-angiogenic therapy was bifurcated into monotherapy with anti-angiogenic drugs and combination therapy with chemotherapy or PARP inhibitors. The corresponding control design was either placebo alone, chemotherapy (alone or plus placebo), or PARP inhibitors only. Notably, the only PARP inhibitor used in the trials was olaparib. Out of the 35 RCTs, 31 were assessed as high quality, whereas 4 were deemed low quality. A notable methodological limitation observed was the lack of double-blinding in the trial design among multiple RCTs (Supplementary Files S2).

**TABLE 1:** The basic characteristics of the included RCTs.

Study ID (trial name/Phase)act	Patients’ status	Drug type	Sample size (E/C)	Median age (E/C, years)	Anti-angiogenic agent treatment group	Control group treatment	Median follow-up duration (E/C, months)	Outcomes in meta-analysis
Coleman 2017 (GOG-0213/Phase 3)	Recurrent, platinum-sensitive, epithelial ovarian, primary peritoneal, or fallopian tube cancer; GOG PS of 0–2	VEGF inhibitor	337/337	59.5/60.6	Pac (175 mg/m^2^) + Carbo (AUC 5) + Bev (15 mg/kg), q3w	Pac (175 mg/m^2^) + Carbo (AUC 5), q3w	49.6	PFS, OS, ORR, Grade ≥3 AEs
Pignata 2021 (MITO16b/MANGO–OV2/ENGOT–ov17/Phase 3)	Platinum-sensitive, FIGO stage IIIB-IV ovarian cancer, fallopian tube carcinoma, or peritoneal carcinoma; ECOG PS of 0–2	VEGF inhibitor	203/203	61/60	Carbo-based doublet + Bev (10 mg/kg intravenous every 14 days)	Carbo-based doublet, i.v	20.1	PFS, OS, ORR, Grade ≥3 AEs
Richardson 2018 (NCT01468909/Phase 2)	Recurrent or persistent epithelial ovarian, fallopian tube, or primary peritoneal cancer; GOG PS of 0–1	VEGFR inhibitor	54/52	61/61	Pac (80 mg/m^2^ on days 1, 8 and 15 every 28 days) + Pazo 800 mg daily	Pac (80 mg/m^2^ on days 1, 8 and 15 every 28 days) + Placebo 800 mg daily	17.7	PFS, OS, ORR
Monk 2016b (TRINOVA-1/Phase 3)	Recurrent partially platinum-sensitive or resistant epithelial ovarian, primary peritoneal or fallopian tube cancers; GOG PS of 0–1	Angiopoietin inhibitor	461/458	60/59	Pac (80 mg/m^2^, days 1, 8, 15, q4w) + Tre (15 mg/kg, qw)	Pac (80 mg/m2, days 1, 8, 15, q4w) + Placebo (15 mg/kg, qw)	18/17.5	PFS, OS, AEs of any grade, Grade ≥3 AEs
Aghajanian 2015 (OCEANS/Phase 3)	Platinum-sensitive, recurrent epithelial ovarian, fallopian tube, or primary peritoneal carcinoma; ECOG PS of 0–1	VEGF inhibitor	242/242	60/61	Cycles 1–6: Gem (1,000 mg/m^2^, days 1 and 8) + Carbo (AUC 4, day 1) + Bev (15 mg/kg on day 1, 6–10 cycles of 21 days); Cycles 10+: Bev (15 mg/kg)	Cycles 1–6: Gem (1,000 mg/m^2^, days 1 and 8) and Carbo (AUC 4, day 1) + Placebo (15 mg/kg on day 1,6–10 cycles of 21 days); Cycles 10+: Placebo (15 mg/kg)	9.6/8.4	OS, AEs of any grade, Grade ≥3 AEs
Karlan 2012 (NCT00479817/Phase 2)	FIGO stage II-IV, recurrent epithelial ovarian, fallopian tube or primary peritoneal cancer; ECOG PS of 0–1	Angiopoietin inhibitor	53 (Tre 3 mg/kg)/53 (Tre 10 mg/kg)/55	60 (Tre 3 mg/kg)/59 (Tre 10 mg/kg)/62	Pac (80 mg/m^2^, days 1, 8, 15, q4w) + Tre (3 mg/kg or 10 mg/kg, qw)	Pac (80 mg/m^2^, days 1, 8, 15, q4w) + Placebo (3 mg/kg or 10 mg/kg, qw)	15.2 (Tre 3 mg/kg)/15.4 (Tre 10 mg/kg)/14.9	PFS, OS, ORR, AEs of any grade, Grade ≥3 AEs
Nicum 2024 (OCTOVA/Phase 2)	Platinum-resistant, relapsed, ovarian, fallopian tube, or primary peritoneal cancer; ECOG PS of ≤2	VEGFR inhibitor	47/46	66/65	Ola (300 mg twice daily) + Ced (20 mg once daily)	Ola (300 mg twice daily)	18	PFS, OS, ORR
Ledermann 2016 (ICON6/Phase 3)	Platinum-sensitive, relapsed, epithelial ovarian cancer, primary peritoneal carcinomatosis or fallopian tube cancer after first-line platinum-based chemotherapy; ECOG PS of 0–1	VEGFR inhibitor	164/118	62/62	Platinum-based chemotherapy + Ced (20 mg, qd) then maintenance Ced (20 mg, qd) alone	Platinum-based chemotherapy + Placebo (20 mg, qd) then maintenance Placebo (20 mg, qd) alone	19.5	PFS
Wang 2022 (APPROVE/Phase 2)	Platinum-resistant, recurrent epithelial ovarian cancer, primary peritoneal cancer, or fallopian tube cancer; ECOG PS of 0–1	VEGFR inhibitor	78/74	54/56	PLD (i.v., 40 mg/m^2^, q4w, up to 6 cycles) + Apa (orally, 250 mg, qd, up to 6 cycles)	PLD (i.v., 40 mg/m^2^, q4w, up to 6 cycles)	8.7	PFS, OS, ORR, AEs of any grade, Grade ≥3 AEs
Shoji 2022 (JGOG3023/Phase 2)	Platinum-resistant, epithelial ovarian, fallopian tube, or primary peritoneal carcinoma; ECOG PS of 0–2	VEGF inhibitor	52/51	60.3 (mean age)/60.7 (mean age)	Chemotherapy (PLD/Topo/Pac/Gem) + Bev (i.v., 15 mg/kg)	Chemotherapy (PLD/Topo/Pac/Gem)	NA	PFS, OS, ORR, AEs of any grade, Grade ≥3 AEs
Gotlieb 2012 (EFC6125/Phase 2)	Platinum-resistant, and Topo-resistant and/or PLD-resistant disease; advanced ovarian cancer patients with recurrent symptomatic malignant ascites; ECOG PS of 0–2	VEGF inhibitor	29/26	60.0/53.5	Afli (i.v., 4.0 mg/kg, q2w)	Placebo (i.v., 4.0 mg/kg, q2w)	NA	OS, AEs of any grade
Marth 2017 (ENGOT-ov-6/TRINOVA-2/Phase 3)	Platinum-resistant epithelial ovarian, peritoneal or fallopian tube cancer; ECOG PS of 0–2	Angiopoietin inhibitor	114/109	61/60	PLD (50 mg/m^2^, q4w) + Tre (15 mg/kg, qw)	PLD (50 mg/m^2^, q4w) + Placebo (15 mg/kg, qw)	12.4	PFS, OS, ORR, AEs of any grade, Grade ≥3 AEs
Pignata 2015 (MITO 11/Phase 2)	Platinum-resistant or refractory ovarian cancer; ECOG PS of 0–1	VEGFR inhibitor	37/36	56/58	Pac (80 mg/m^2^ on days 1, 8 and 15 in every 28 days) + Pazo (800 mg daily)	Pac (80 mg/m^2^ on days 1, 8 and 15 every 28 days)	16.3/16.1	PFS, OS, ORR, Grade ≥3 AEs
Chekerov 2018 (TRIAS/Phase 2)	Platinum-resistant ovarian, peritoneal, or fallopian tube cancers; ECOG PS of 0–2	VEGFR inhibitor	83/89	59/58	Cycles 1–6: Topo (1–25 mg/m^2^ on days 1–5) + Sor (400 mg oral bid on days 6–15, every 21 days); Cycles 6+: Daily maintenance Sor for up to 1 year	Cycles 1–6: Topo (1–25 mg/m^2^ on days 1–5) + Placebo (bid on days 6–15, every 21 days); Cycles 6+: Daily maintenance Placebo for up to 1 year	11.3/8.7	PFS, OS, ORR, AEs of any grade, Grade ≥3 AEs
Liu 2019 (NCT01116648/Phase 2)	Relapsed platinum-sensitive ovarian cancer of high-grade serous or endometrioid histology or had a deleterious germline BRCA1/2 mutation	VEGFR inhibitor	44/46	58.1/57.8	Ola (200 mg, bid) + Ced (30 mg daily)	Ola (400 mg, bid)	46	PFS, OS
Pujade-Lauraine 2014 (AURELIA/Phase 3)	Platinum-resistant, recurrent epithelial ovarian, fallopian tube or primary peritoneal cancer; ECOG PS of 0–2	VEGF inhibitor	179/182	62/61	Chemotherapy (PLD/Pac/Topo) + Bev (15 mg/kg, q3w or 10 mg/kg, q2w)	Chemotherapy (PLD/Pac/Topo)	13.0/13.9	PFS, OS, ORR
Liu 2022 (NRG-GY004/Phase 3)	Platinum-sensitive, relapsed high-grade serous or high-grade endometrioid ovarian, primary peritoneal, or fallopian tube cancer	VEGFR inhibitor	189/189	NA	Ola (200 mg tablets, bid) + Ced (30 mg tablet, qd)	Ola (300 mg tablets, bid)	24 (mean duration)	PFS, ORR
Ledermann 2021 (ICON6/Phase 3)	Platinum-sensitive, relapsed, epithelial ovarian cancer, primary peritoneal carcinomatosis or fallopian tube cancer after first-line platinum-based chemotherapy; ECOG PS of 0–1	VEGFR inhibitor	164/118	62/62	Platinum-based chemotherapy + Ced (20 mg, qd) then maintenance Ced (20 mg, qd) alone	Platinum-based chemotherapy + Placebo (20 mg, qd) then maintenance Placebo (20 mg, qd) alone	25.6	OS
Ferron 2023 (GINECO/Phase 2)	Newly diagnosed epithelial ovarian, fallopian tube, or primary peritoneal cancer; FIGO stage IIIC/IV, and ECOG PS of ≤2	VEGFR inhibitor	124/64	64/63.5	Nin (200 mg, bid, on days 2–21, q3w, for up to 2 years)	Placebo (bid, on days 2–21, q3w, for up to 2 years)	42.6	PFS, OS, ORR, AEs of any grade, Grade ≥3 AEs
Burger 2011 (GOG-0218/Phase 3)	Newly diagnosed, FIGO stage III or IV epithelial ovarian, primary peritoneal or fallopian tube cancer; GOG PS of 0–2	VEGF inhibitor	623/625	60/60	Cycles 1–6: Pac (175 mg/m^2^) + Carbo (AUC 6) + Bev (15 mg/kg), q3w; Cycles 7–22: Bev (15 mg/kg), q3w	Cycles1-6: Pac (175 mg/m^2^) + Carbo (AUC 6) + Placebo, q3w; Cycles 7–22: Placebo,q3w	17.4	PFS
Aghajanian 2012 (OCEANS/Phase 3)	Platinum-sensitive, recurrent epithelial ovarian, fallopian tube, or primary peritoneal carcinoma; ECOG PS of 0–1	VEGF inhibitor	242/242	60.5/61.6	Cycles 1–10: Gem (1,000 mg/m^2^ on days 1 and 8) + Carbo (AUC 4 on day 1) + Bev (15 mg/kg on day 1), q3w	Cycles 1–10: Gem (1,000 mg/m^2^, days 1 and 8) + Carbo (AUC 4, day 1) + Placebo (15 mg/kg, day 1), q3w	24	PFS, ORR
Oza 2015 (ICON7/Phase 3)	FIGO stage I-IIA newly diagnosed ovarian cancer or more FIGO stage IIB-IV advanced disease	VEGF inhibitor	764/764	57	Cycles 1–6: Pac (175 mg/m^2^) + Carbo (AUC 5 or 6), q3w + Bev (7.5 mg/kg, q3w); Cycles 7–18: Bev (7.5 mg/kg, q3w)	Cycles 1–6: Pac (175 mg/m^2^) + Carbo (AUC 5 or 6), q3w	48.8/48.6	PFS, OS
du Bois 2016 (AGO-OVAR 12/Phase 3)	Chemotherapy-naive, FIGO stage IIB-IV, epithelial ovarian cancer, fallopian tube or primary peritoneal cancer; ECOG PS of 0–2	VEGFR inhibitor	911/455	58/58	Cycles1-6: Pac (175 mg/m^2^) + Carbo (AUC 5 or 6) + Nin (200 mg, bid, days 2–21, q3w) followed by Nin maintenance	Cycles1-6: Pac (175 mg/m^2^) + Carbo (AUC 5 or 6) + Placebo (200 mg, bid, days 2–21, q3w) followed by Placebo maintenance	18	AEs of any grade, Grade ≥3 AEs
Ledermann 2011 (NCT00710762/Phase 2)	Advanced ovarian carcinoma, fallopian tube carcinoma or primary peritoneal cancer of serous type with recurrent disease and who responded to second-, third-, or fourth-line chemotherapy; ECOG PS of 0–1	VEGFR inhibitor	43/40	60/63	Cycles 1–9: Nin (250 mg, bid, q4w)	Cycles 1–9: Placebo (250 mg, bid, q4w)	36 weeks (follow-up endpoint)	PFS, OS, Grade ≥3 AEs
du Bois 2014 (NCT00866697/Phase 3)	FIGO stage II-IV, epithelial ovarian, fallopian tube or primary peritoneal carcinoma who have not progressed after first-line chemotherapy; ECOG PS of 0–1	VEGFR inhibitor	472/468	56/57	Pazo (800 mg, orally, qd, for up to 24 months)	Placebo (800 mg, orally, qd, for up to 24 months)	24.3	PFS, OS
Herzog 2013 (NCT00791778/Phase 2)	FIGO stage III-IV ovarian epithelial cancer or primary peritoneal cancer who have achieved a response after standard platinum/taxane containing chemotherapy (first-line therapy); ECOG PS of 0–1	VEGFR inhibitor	123/123	56.9/54.4	Sor (400 mg, orally, bid, every 12 h)	Placebo (400 mg, orally, bid, every 12 h)	NA	PFS, OS
Tewari 2019 (GOG-0218/Phase 3)	Newly diagnosed ovarian, fallopian tube, or primary peritoneal carcinoma	VEGF inhibitor	623/625	60/60	Cycles 1–6: Pac (175 mg/m^2^) + Carbo (AUC 6) + Bev (15 mg/kg, cycle 2 +) every 21 days; Cycles 7–22: Bev maintenance (15 mg/kg) every 21 days	Cycles 1–6: Pac (175 mg/m^2^) + Carbo (AUC 6) + Placebo (cycle 2+) every 21 days; Cycles 7–22: Placebo every 21 days	101.9/103.4	OS
Vergote 2019a (AGO-OVAR16/Phase 3)	FIGO stage II-IV epithelial ovarian, fallopian tube, or primary peritoneal carcinoma	VEGFR inhibitor	472/468	56/57	Pazo (800 mg, qd, for up to 24 months)	Placebo (800 mg, qd, for up to 24 months)	NA	OS
Kim 2018 (East Asian study/Phase 3)	Advanced ovarian, fallopian tube or primary peritoneal carcinoma	VEGFR inhibitor	73/72	51.7/54.1	Pazo (800 mg, qd, for up to 24 months)	Placebo (800 mg, qd, for up to 24 months)	NA	PFS, AEs of any grade, Grade ≥3 AEs
Ray-Coquard 2020 (AGO-OVAR 12/Phase 3)	FIGO stage IIB-IV newly diagnosed advanced epithelial ovarian, fallopian tube or primary peritoneal cancer	VEGFR inhibitor	911/455	58/58	Nin (200 mg, bid, on days 2–21, every 21 days) + Pac (175 mg/m^2^) + Carbo (AUC 5 or 6, day 1, every 21 days for six cycles)	Placebo (200 mg, bid, on days 2–21, every 21 days) + Pac (175 mg/m^2^) + Carbo (AUC 5 or 6, day 1, every 21 days for six cycles)	60.9	PFS, OS
Vergote 2019b (TRINOVA-3/Phase 3)	FIGO stage III-IV epithelial ovarian, primary peritoneal, or fallopian tube cancer; ECOG PS of 0–1	Angiopoietin inhibitor	678/337	59/59	Cycles 1–6: Pac (175 mg/m^2^) + Carbo (AUC 5 or 6, every 3 weeks) + Tre (15 mg/kg); Cycles 6+: Tre for up to 18 additional months	Cycles 1–6: Pac (175 mg/m^2^) + Carbo (AUC 5 or 6, every 3 weeks) + Placebo (15 mg/kg); Cycles 6+: Placebo for up to 18 additional months	27.4	PFS, OS, AEs of any grade, Grade ≥3 AEs
Duska 2020 (NCT01610206/Phase 2)	Persistent or recurrent epithelial ovarian, fallopian tube or primary peritoneal carcinoma	VEGFR inhibitor	75/73	63	Gem (1,000 mg/m^2^, weekly on days 1 and 8, every 21 days + Pazo (800 mg, orally, daily)	Gem (1,000 mg/m^2^, weekly on days 1 and 8, every 21 days	13	PFS, ORR
Roque 2022 (NCT03093155/Phase 2)	Platinum-resistant or refractory epithelial (non-mucinous) ovarian, fallopian tube, or primary peritoneal carcinoma; ECOG PS of 0–2	VEGF inhibitor	39/37	67/67	Ixa (20 mg/m^2^, i.v., days 1, 8, and 15 of a 28-day cycle) + Bev (10 mg/kg, i.v., days 1, 15 every 28 days)	Ixa (20 mg/m^2^, i.v., days 1, 8, and 15 of a 28-day cycle)	NA	PFS, OS, ORR
Hall 2020 (NCT01610869/Phase 2)	Platinum resistant or intolerant ovarian, fallopian tube or primary peritoneal carcinoma	VEGFR inhibitor	59/55	62.4/65.7	Cyc (orally, 100 mg, qd, in cycles of 6 weeks) + Nin (200 mg, bid)	Cyc (orally, 100 mg, qd, in cycles of 6 weeks)	19.2	PFS, OS, ORR, AEs of any grade, Grade ≥3 AEs
Gore 2019 (mEOC/GOG 0241/Phase 3)	FIGO stage II-IV primary mucinous epithelial ovarian cancer or recurrence after stage I disease	VEGF inhibitor	24/26	47; 51/55; 56	Pac (175 mg/m^2^) + Carbo (AUC5 or 6) + Bev (15 mg/kg, 3-weekly maintenance, 12cycles); Oxa (130 mg/m^2^) + Cap (850 mg/m^2^, bid, days 1–14) + Bev (15 mg/kg, 3-weekly maintenance, 12 cycles)	Pac (175 mg/m^2^) + Carbo (AUC 5 or 6); Oxa (130 mg/m^2^) + Cap (850 mg/m^2^, bid, days 1–14)	59	PFS, OS, ORR, Grade ≥3 AEs

E, experimental group; C, control group; GOG, the Gynecologic Oncology Group; PS, performance status; VEGF, vascular endothelial growth factor; Pac, paclitaxel; Carbo, carboplatin; AUC, area under curve; Bev, bevacizumab; q3w, every 3 weeks; PFS, progression-free survival; OS, overall survival; ORR, objective response rate; AEs, adverse events; FIGO, international federation of gynecology and obstetrics; ECOG, eastern cooperative oncology group; VEGFR, vascular endothelial growth factor receptor; Pazo, pazopanib; Tre, trebananib; Gem, gemcitabine; Ola, olaparib; Ced, cediranib; qd, once daily; PLD, pegylated liposomal doxorubicin; i.v., intravenously; Apa, Apatinib; NA, not available; Afli, aflibercept; Topo, topotecan; Sor, sorafenib; bid, twice daily; Nin, nintedanib; Ixa, ixabepilone; Cyc, cyclophosphamide; Oxa, oxaliplatin; Cap, capecitabine.

### 3.3 Overall analysis of anti-angiogenic drug monotherapy

**FIGURE 2:**
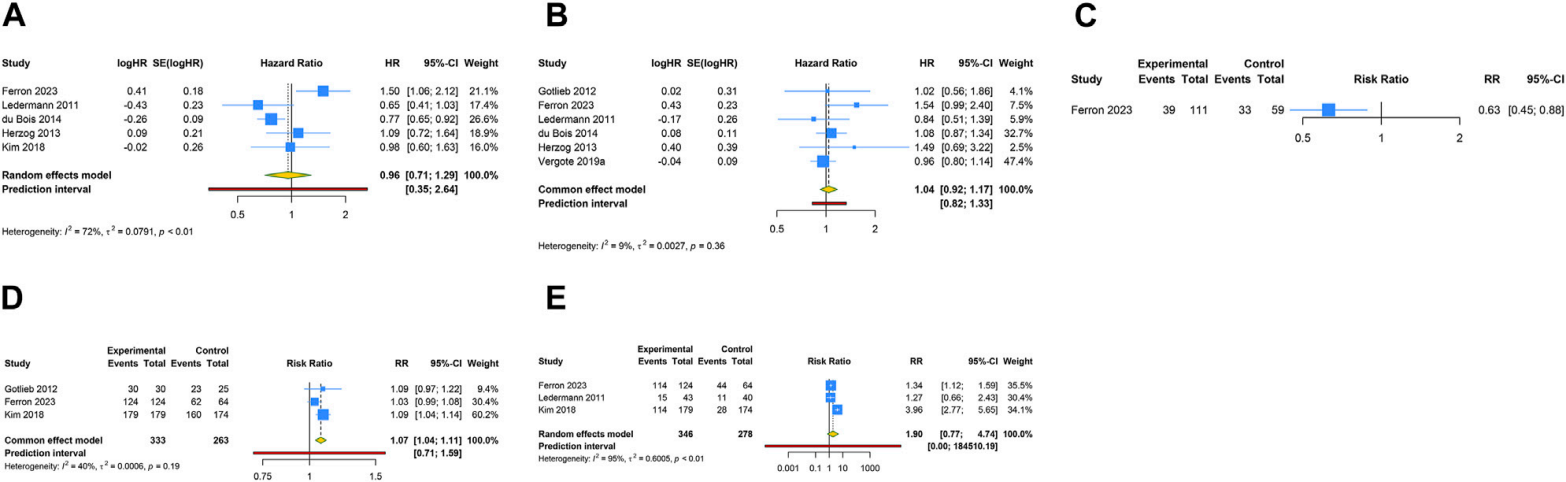
Forest plot of the efficacy and safety outcomes after anti-angiogenic agent monotherapy for ovarian cancer. **(A)** Progression-free survival; **(B)** Overall survival; **(C)** Objective response rate; **(D)** Adverse events of any grade; **(E)** Grade ≥3 adverse events.

5 RCTs were conducted to evaluate the PFS benefit of anti-angiogenic drug monotherapy in OC patients. Owing to substantial heterogeneity observed across these trials, a random-effects model was employed for analysis (I^2^ = 72.1%, Tau^2^ = 0.0791). The combined estimate indicated that anti-angiogenic monotherapy did not provide a significant PFS advantage over placebo (HR [95% CI] = 0.956 [0.709–1.288], 95% PI: 0.345–2.645). Similarly, the consolidated results from a fixed-effects model (I^2^ = 8.6%, Tau^2^ = 0.0027), derived from 6 RCTs, revealed that anti-angiogenic drug monotherapy did not significantly enhance OS (HR [95% CI] = 1.039 [0.921–1.173], 95% PI: 0.824–1.331). A single study reported on the ORR associated with monotherapy (Ferron et al., 2023), revealing a lower ORR with the use of anti-angiogenic monotherapy (specifically nintedanib) as compared to placebo (RR [95% CI] = 0.628 [0.447–0.882]). Concerning AEs, pooled results from 3 trials suggested that the incidence of any grade AEs was significantly higher with anti-angiogenic monotherapy compared to placebo (RR [95% CI] = 1.072 [1.036–1.109], 95% PI: 0.709–1.592; I^2^ = 40.1%, Tau^2^ = 0.0006). However, there was no significant difference in the risk of grade ≥3 AEs between the monotherapy group and the control group (RR [95% CI] = 1.905 [0.766–4.736]; I^2^ = 95.0%, Tau^2^ = 0.6005) (Table 2; Figure 2).

**TABLE 2:** Pooled effect of the efficacy and safety of monotherapy or combination therapy with anti-angiogenic drugs in the treatment of ovarian cancer.

Outcomes	Number of studies	Meta-analysis				Heterogeneity	
		HR/RR	95% CI	*p*-value	95% PI	I^2^, Tau^2^	*p*-value
Antiangiogenic agent monotherapy vs Placebo							
PFS	5	0.956	0.709–1.288	0.766	0.345–2.645	72.1%, 0.0791	0.006
OS	6	1.039	0.921–1.173	0.532	0.824–1.331	8.6%, 0.0027	0.361
ORR	1	0.628	0.447–0.882	0.007			
AEs of any grade	3	1.072	1.036–1.109	<0.001	0.709–1.592	40.1%, 0.0006	0.188
Grade ≥3 AEs	3	1.905	0.766–4.736	0.166	-	95.0%, 0.6005	<0.001
Antiangiogenic agents + Other drugs vs Other drugs (alone or + Placebo)							
PFS	24	0.678	0.606–0.759	<0.001	0.415–1.108	79.5%, 0.0529	<0.001
OS	23	0.917	0.870–0.966	0.001	0.851–0.984	2.6%, 0.0005	0.425
ORR	18	1.441	1.287–1.614	<0.001	1.032–2.014	52.1%, 0.0216	0.005
AEs of any grade	11	1.011	0.999–1.022	0.069	0.980–1.043	56.6%, 0.0002	0.011
Grade ≥3 AEs	15	1.137	1.099–1.177	<0.001	1.011–1.252	33.4%, 0.0019	0.101

PFS, progression-free survival; OS, overall survival; ORR, objective response rate; AEs, adverse events.

### 3.4 Overall analysis of anti-angiogenic drug combination therapy

**FIGURE 3:**
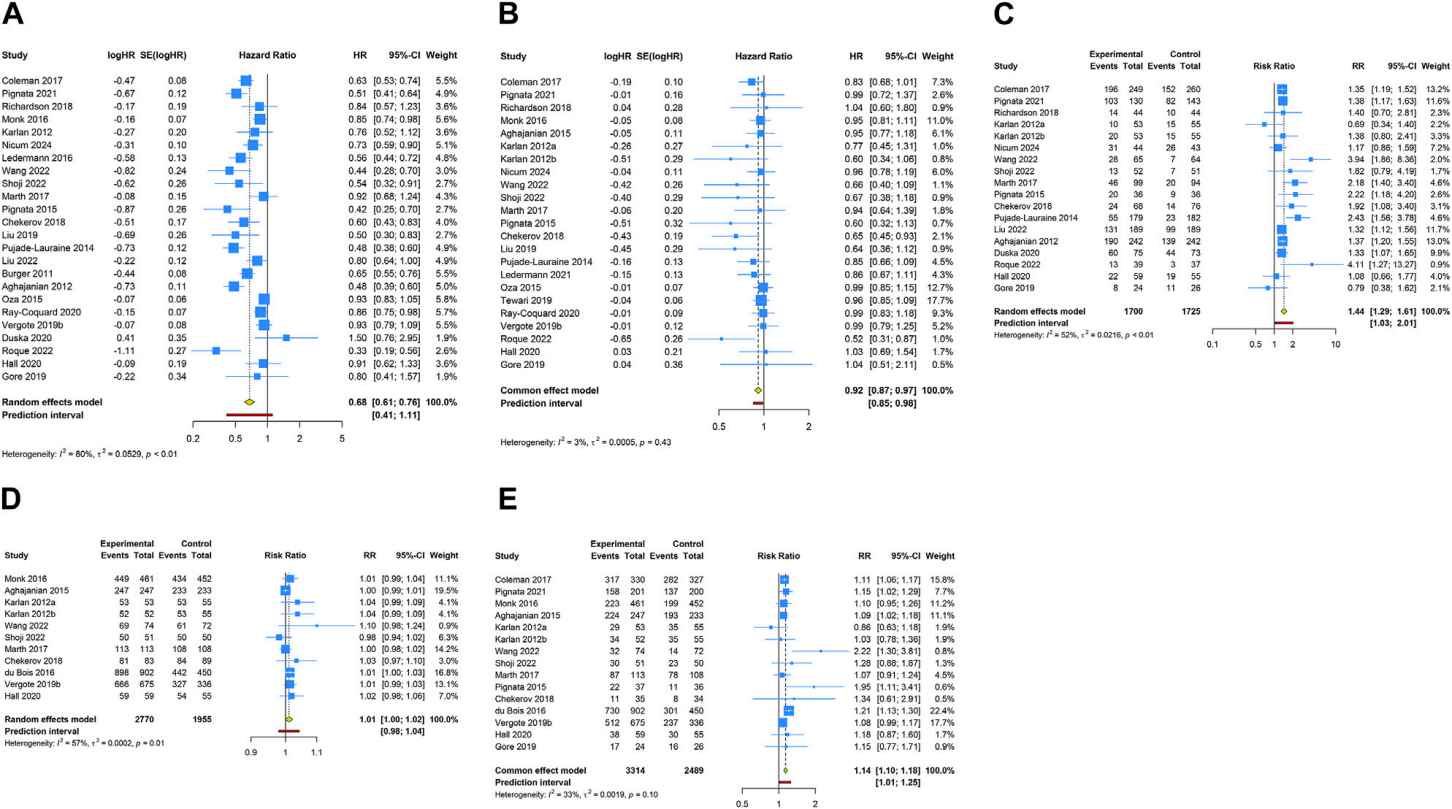
Forest plot of the efficacy and safety outcomes after anti-angiogenic drug combination therapy for ovarian cancer. **(A)** Progression-free survival; **(B)** Overall survival; **(C)** Objective response rate; **(D)** Adverse events of any grade; **(E)** Grade ≥3 adverse events.

A total of 24 RCTs evaluated PFS advantage of anti-angiogenic drug combination therapy in patients with OC. Given the notable heterogeneity in the studies regarding PFS, a random-effects model was utilized for the pooled PFS analysis (I^2^ = 79.5%, Tau^2^ = 0.0529). The overall analysis revealed that the combination therapy of anti-angiogenic drugs led to a 32.2% decrease in the risk of disease progression or death when contrasted with regimens excluding anti-angiogenic drugs (HR [95% CI] = 0.678 [0.606–0.759], 95% PI: 0.415–1.108). Likewise, the pooled findings from a fixed-effects model (I^2^ = 2.6%, Tau^2^ = 0.0005), based on 23 RCTs, demonstrated a significant improvement in OS when anti-angiogenic drugs were used in combination therapy compared with the control (HR [95% CI] = 0.917 [0.870–0.966], 95% PI: 0.851–0.984). Furthermore, 18 studies reported the ORR outcome of combination therapy, and the results showed that the ORR of anti-angiogenic agents combined with other drugs was significantly higher than that of other drugs alone (RR [95% CI] = 1.441 [1.287–1.614], 95% PI: 1.032–2.014; I^2^ = 52.1%, Tau^2^ = 0.0216). Regarding AEs, the consolidated results from 11 trials indicated no significant difference in the risk of any grade AEs between the combination therapy group and the control group (RR [95% CI] = 1.011 [0.999–1.022], 95% PI: 0.980–1.043; I^2^ = 56.6%, Tau^2^ = 0.0002). However, the occurrence of grade ≥3 AEs was significantly increased in the combination therapy group compared to the control group (RR [95% CI] = 1.137 [1.099–1.177], 95% PI: 1.011–1.252; I^2^ = 33.4%, Tau^2^ = 0.0019) (Table 2; Figure 3).

### 3.5 Subgroup analysis of anti-angiogenic drug monotherapy

Subgroup analyses were conducted only for categories comprising two or more studies. When stratified by OC subtype, it was observed that anti-angiogenic drug monotherapy escalated the risk of any grade AEs in patients with advanced OC, relative to placebo (RR [95% CI] = 1.072 [1.036–1.109], 95% PI: 0.709–1.592; I^2^ = 40.1%, Tau^2^ = 0.0006). Yet, in the context of advanced OC, no significant impact on PFS, OS, or the occurrence of grade ≥ 3AEs was observed with anti-angiogenic drug monotherapy (all *p* > 0.05). Further, stratified analyses predicated on the classification of anti-angiogenic drugs revealed an increased incidence of any grade AEs with VEGFR inhibitors compared to placebo (RR [95% CI] = 1.059 [1.003–1.119]; I^2^ = 68.2%, Tau^2^ = 0.0011). Subsequent analysis grouped by specific anti-angiogenic agents suggested that pazopanib significantly improved PFS (HR [95% CI] = 0.791 [0.670–0.934]; I^2^ = 0%, Tau^2^ = 0), while nintedanib was associated with a higher incidence of grade ≥3 AEs (RR [95% CI] = 1.326 [1.109–1.586]; I^2^ = 0%, Tau^2^ = 0). The complete results of the subgroup analysis were detailed in Table 3 and Supplementary Figure S1–S4.

**TABLE 3:** Subgroup analysis of the efficacy and safety of anti-angiogenic agent monotherapy for ovarian cancer.

Outcomes and subgroups	Number of studies	Meta-analysis			95% PI	Heterogeneity	
		HR/RR	95% CI	*p*-value		I^2^, Tau^2^	*p*-value
PFS							
Subgrouped by ovarian cancer subtypes							
Advanced ovarian cancer	3	1.003	0.603–1.671	0.99	0.003–396.633	76.2%, 0.1538	0.015
Subgrouped by types of anti-angiogenic drugs							
VEGFR inhibitors vs Placebo	5	0.956	0.709–1.288	0.766	0.345–2.645	72.1%, 0.0791	0.006
Nintedanib vs Placebo	2	1.001	0.441–2.271	0.998	-	87.9%, 0.3072	0.004
Pazopanib vs Placebo	2	0.791	0.670–0.934	0.006	-	0%, 0	0.366
OS							
Subgrouped by ovarian cancer subtypes							
Advanced ovarian cancer	4	1.179	0.899–1.548	0.234	0.474–2.911	19.6%, 0.0197	0.292
Subgrouped by types of anti-angiogenic drugs							
VEGFR inhibitors vs Placebo	5	1.04	0.919–1.177	0.532	0.707–1.597	26.8%, 0.0093	0.243
Nintedanib vs Placebo	2	1.151	0.636–2.084	0.643	-	68.3%, 0.1255	0.076
Pazopanib vs Placebo	2	1.007	0.880–1.154	0.917	-	0%, 0	0.403
AEs of any grade							
Subgrouped by ovarian cancer subtypes							
Advanced ovarian cancer	3	1.072	1.036–1.109	<0.001	0.709–1.592	40.1%, 0.0006	0.188
Subgrouped by types of anti-angiogenic drugs							
VEGFR inhibitors vs Placebo	2	1.059	1.003–1.119	0.04	-	68.2%, 0.0011	0.076
Grade ≥3 AEs							
Subgrouped by ovarian cancer subtypes							
Advanced ovarian cancer	3	1.905	0.766–4.736	0.166	-	95.0%, 0.6005	<0.001
Subgrouped by types of anti-angiogenic drugs							
VEGFR inhibitors vs Placebo	3	1.905	0.766–4.736	0.166	-	95.0%, 0.6005	<0.001
Nintedanib vs Placebo	2	1.326	1.109–1.586	0.002	-	0%, 0	0.869

PFS, progression-free survival; OS, overall survival; AEs, adverse events.

### 3.6 Subgroup analysis of anti-angiogenic drug combination therapy

Subgroup analyses were carried out solely for groups that included two or more studies. Categorized by OC subtypes, it was observed that anti-angiogenic drug combination therapy significantly improved PFS compared with drug therapy without anti-angiogenic agents in patients with platinum-sensitive and recurrent OC (HR [95% CI] = 0.612 [0.519–0.722], 95% PI: 0.355–1.055; I^2^ = 78.5%, Tau^2^ = 0.0460), platinum-resistant OC (HR [95% CI] = 0.691 [0.494–0.966], 95% PI: 0.019–25.494; I^2^ = 59.4%, Tau^2^ = 0.0514), newly diagnosed OC (HR [95% CI] = 0.807 [0.657–0.990], 95% PI: 0.066–9.808; I^2^ = 85.3%, Tau^2^ = 0.0278), and platinum-resistant or refractory OC (HR [95% CI] = 0.374 [0.259–0.540]; I^2^ = 0%, Tau^2^ = 0). Similarly, it was noted that combination therapy with anti-angiogenic drugs was associated with a significant improvement in OS among patients with platinum-sensitive and recurrent OC (HR [95% CI] = 0.892 [0.822–0.968], 95% PI: 0.806–0.988; I^2^ = 0%, Tau^2^ = 0), platinum-resistant OC (HR [95% CI] = 0.753 [0.592–0.956], 95% PI: 0.146–3.886; I^2^ = 2.6%, Tau^2^ = 0.0013), and platinum-resistant or refractory OC (HR [95% CI] = 0.551 [0.369–0.821]; I^2^ = 0%, Tau^2^ = 0). Moreover, the combined therapeutic approach of anti-angiogenic drugs exhibited a comparatively high ORR for patients with platinum-sensitive and recurrent OC, platinum-resistant OC, recurrent or persistent OC, platinum-resistant or refractory OC, and advanced OC (all *p* < 0.05). However, it is important to note that for individuals with advanced OC, combination therapy with anti-angiogenic drugs can also lead to a higher incidence of any grade AEs (RR [95% CI] = 1.014 [1.003–1.025], 95% PI: 0.948–1.085; I^2^ = 0%, Tau^2^ = 0) and grade ≥3 AEs (RR [95% CI] = 1.151 [1.097–1.209], 95% PI: 0.921–1.428; I^2^ = 34.2%, Tau^2^ = 0.0015). Particularly, patients with platinum-sensitive and recurrent OC receiving combination therapy experienced an elevated frequency of grade ≥3 AEs (RR [95% CI] = 1.120 [1.036–1.210], 95% PI: 0.836–1.500; I^2^ = 57.8%, Tau^2^ = 0.0031) (Table 4; Supplementary Figure S5–S9).

**TABLE 4:** Subgroup analysis of the efficacy and safety of anti-angiogenic agent combination therapy for ovarian cancer.

Outcomes and subgroups	Number of studies	Meta-analysis			95% PI	Heterogeneity	
		HR/RR	95% CI	*p*-value		I^2^, Tau^2^	*p*-value
PFS							
Subgrouped by ovarian cancer subtypes							
Platinum-sensitive and recurrent ovarian cancer	9	0.612	0.519–0.722	<0.001	0.355–1.055	78.5%, 0.0460	<0.001
Platinum-resistant ovarian cancer	3	0.691	0.494–0.966	0.031	0.019–25.494	59.4%, 0.0514	0.085
Newly diagnosed ovarian cancer	3	0.807	0.657–0.990	0.039	0.066–9.808	85.3%, 0.0278	0.001
Recurrent or persistent ovarian cancer	3	0.872	0.678–1.120	0.283	0.048–16.920	33.4%, 0.0269	0.223
Platinum-resistant or refractory ovarian cancer	2	0.374	0.259–0.540	<0.001	-	0%, 0	0.52
Advanced ovarian cancer	3	0.752	0.493–1.146	0.185	0.004–135.898	89.1%, 0.1210	<0.001
Subgrouped by types of anti-angiogenic drugs							
VEGF inhibitors + CT vs CT (alone or + PL)	9	0.580	0.470–0.715	<0.001	0.286–1.175	86.7%, 0.0779	<0.001
Bevacizumab + CT vs CT (alone or + PL)	9	0.580	0.470–0.715	<0.001	0.286–1.175	86.7%, 0.0779	<0.001
VEGFR inhibitors + Other drugs vs Other drugs (alone or + PL)	11	0.697	0.595–0.818	<0.001	0.426–1.143	66.0%, 0.0410	0.001
Pazopanib + CT vs CT (alone or + PL)	3	0.786	0.415–1.490	0.461	-	78.7%, 0.2475	0.009
Cediranib + Other drugs vs Other drugs (alone or + PL)	4	0.669	0.552–0.810	<0.001	0.324–1.381	51.5%, 0.0189	0.103
Nintedanib + CT vs CT (alone or + PL)	2	0.865	0.763–0.982	0.025	-	0%, 0	0.782
Angiopoietin inhibitors + CT vs PL + CT	4	0.879	0.798–0.968	0.009	0.711–1.087	0%, 0	0.722
Trebananib + CT vs PL + CT	4	0.879	0.798–0.968	0.009	0.711–1.087	0%, 0	0.722
OS							
Subgrouped by ovarian cancer subtypes							
Platinum-sensitive and recurrent ovarian cancer	8	0.892	0.822–0.968	0.006	0.806–0.988	0%, 0	0.668
Platinum-resistant ovarian cancer	3	0.753	0.592–0.956	0.02	0.146–3.886	2.6%, 0.0013	0.358
Newly diagnosed ovarian cancer	3	0.977	0.899–1.061	0.575	0.570–1.673	0%, 0	0.937
Recurrent or persistent ovarian cancer	3	0.788	0.574–1.083	0.142	0.101–6.159	0%, 0	0.391
Platinum-resistant or refractory ovarian cancer	2	0.551	0.369–0.821	0.003	-	0%, 0	0.731
Advanced ovarian cancer	3	0.997	0.841–1.181	0.972	0.332–2.994	0%, 0	0.985
Subgrouped by types of anti-angiogenic drugs							
VEGF inhibitors + CT vs CT (alone or + PL)	9	0.923	0.859–0.991	0.028	0.791–1.061	13.5%, 0.0021	0.322
Bevacizumab + CT vs CT (alone or + PL)	9	0.923	0.859–0.991	0.028	0.791–1.061	13.5%, 0.0021	0.322
VEGFR inhibitors + Other drugs vs Other drugs (alone or + PL)	9	0.895	0.809–0.990	0.031	0.690–1.112	19.2%, 0.0064	0.272
Pazopanib + Paclitaxel vs Paclitaxel (alone or + PL)	2	0.822	0.544–1.242	0.351	-	40.1%, 0.0606	0.197
Cediranib + Other drugs vs Other drugs (alone or + PL)	3	0.892	0.763–1.043	0.152	0.323–2.461	0%, 0	0.392
Nintedanib + CT vs CT (alone or + PL)	2	0.996	0.850–1.167	0.960	-	0%, 0	0.860
Angiopoietin inhibitors + CT vs PL + CT	5	0.931	0.828–1.047	0.235	0.770–1.127	0%, 0	0.538
Trebananib + CT vs PL + CT	5	0.931	0.828–1.047	0.235	0.770–1.127	0%, 0	0.538
ORR							
Subgrouped by ovarian cancer subtypes							
Platinum-sensitive and recurrent ovarian cancer	6	1.454	1.237–1.710	<0.001	0.898–2.356	70.0%, 0.0234	0.005
Platinum-resistant ovarian cancer	3	2.034	1.473–2.809	<0.001	0.252–16.525	0%, 0	0.901
Recurrent or persistent ovarian cancer	4	1.235	1.008–1.512	0.042	0.698–2.288	9.1%, 0.0065	0.348
Platinum-resistant or refractory ovarian cancer	2	2.704	1.535–4.763	0.001	-	0%, 0	0.355
Advanced ovarian cancer	2	1.321	1.122–1.554	0.001	-	0%, 0	0.335
Subgrouped by types of anti-angiogenic drugs							
VEGF inhibitors + CT vs CT (alone or + PL)	7	1.441	1.241–1.674	<0.001	0.985–2.109	55.2%, 0.0161	0.037
Bevacizumab + CT vs CT (alone or + PL)	7	1.441	1.241–1.674	<0.001	0.985–2.109	55.2%, 0.0161	0.037
VEGFR inhibitors + Other drugs vs Other drugs (alone or + PL)	8	1.444	1.191–1.752	<0.001	0.874–2.389	50.8%, 0.0325	0.047
Pazopanib + CT vs CT (alone or + PL)	3	1.465	1.186–1.811	<0.001	0.139–15.216	18.4%, 0.0150	0.294
Cediranib + Olaparib vs Olaparib	2	1.290	1.115–1.493	<0.001	-	0%, 0	0.476
Angiopoietin inhibitors + CT vs PL + CT	3	1.342	0.719–2.505	0.355	0.001–1810.780	73.2%, 0.2204	0.024
Trebananib + CT vs PL + CT	3	1.342	0.719–2.505	0.355	0.001–1810.780	73.2%, 0.2204	0.024
AEs of any grade							
Subgrouped by ovarian cancer subtypes							
Platinum-sensitive and recurrent ovarian cancer	3	1.018	0.971–1.068	0.463	0.586–1.768	88.9%, 0.0013	<0.001
Platinum-resistant ovarian cancer	3	1.007	0.983–1.033	0.56	0.780–1.280	38.7%, 0.0002	0.196
Recurrent ovarian cancer	2	1.037	0.992–1.084	0.107	-	0%, 0	0.982
Advanced ovarian cancer	3	1.014	1.003–1.025	0.014	0.948–1.085	0%, 0	0.971
Subgrouped by types of anti-angiogenic drugs							
VEGF inhibitors + CT vs CT (alone or + PL)	2	0.997	0.985–1.008	0.58	-	23.7%, <0.0001	0.252
Bevacizumab + CT vs CT (alone or + PL)	2	0.997	0.985–1.008	0.58	-	23.7%, <0.0001	0.252
VEGFR inhibitors + CT vs CT (alone or + PL)	4	1.023	1.007–1.039	0.004	0.965–1.075	15.9%, <0.0001	0.312
Nintedanib + CT vs CT (alone or + PL)	2	1.014	1.001–1.027	0.032	-	0%, 0	0.809
Angiopoietin inhibitors + CT vs PL + CT	5	1.015	1.002–1.029	0.03	0.991–1.031	2.7%, <0.0001	0.391
Trebananib + CT vs PL + CT	5	1.015	1.002–1.029	0.03	0.991–1.031	2.7%, <0.0001	0.391
Grade ≥3 AEs							
Subgrouped by ovarian cancer subtypes							
Platinum-sensitive and recurrent ovarian cancer	4	1.12	1.036–1.210	0.004	0.836–1.500	57.8%, 0.0031	0.069
Platinum-resistant ovarian cancer	3	1.13	0.973–1.313	0.11	0.443–2.740	0%, 0	0.566
Recurrent ovarian cancer	2	0.943	0.764–1.164	0.586	-	0%, 0	0.408
Advanced ovarian cancer	4	1.151	1.097–1.209	<0.001	0.921–1.428	34.2%, 0.0015	0.207
Subgrouped by types of anti-angiogenic drugs							
VEGF inhibitors + CT vs CT (alone or + PL)	5	1.122	1.075–1.170	<0.001	1.048–1.184	0%, 0	0.885
Bevacizumab + CT vs CT (alone or + PL)	5	1.122	1.075–1.170	<0.001	1.048–1.184	0%, 0	0.885
VEGFR inhibitors + CT vs CT (alone or + PL)	5	1.259	1.172–1.352	<0.001	0.720–2.667	49.8%, 0.0291	0.093
Nintedanib + CT vs CT (alone or + PL)	2	1.208	1.126–1.296	<0.001	-	0%, 0	0.879
Angiopoietin inhibitors + CT vs PL + CT	5	1.068	1.002–1.138	0.045	0.966–1.178	0%, 0	0.724
Trebananib + CT vs PL + CT	5	1.068	1.002–1.138	0.045	0.966–1.178	0%, 0	0.724

PFS, progression-free survival; CT, chemotherapy; PL, placebo; OS, overall survival; ORR, objective response rate; AEs, adverse events.

Subgroup analysis according to the types of anti-angiogenic drugs indicated that VEGF inhibitors combined with chemotherapy significantly improved PFS (HR [95% CI] = 0.580 [0.470–0.715], 95% PI: 0.286–1.175; I^2^ = 86.7%, Tau^2^ = 0.0779) and OS (HR [95% CI] = 0.923 [0.859–0.991], 95% PI: 0.791–1.061; I^2^ = 13.5%, Tau^2^ = 0.0021), and also increased the ORR (RR [95% CI] = 1.441 [1.241–1.674], 95% PI: 0.985–2.109; I^2^ = 55.2%, Tau^2^ = 0.0161) and the risk of grade ≥3 AEs (RR [95% CI] = 1.122 [1.075–1.170], 95% PI: 1.048–1.184; I^2^ = 0%, Tau^2^ = 0) compared with chemotherapy alone or with placebo. These results were replicated in the combination therapy with bevacizumab. In addition, combination therapy with VEGFR inhibitors was found to be associated with improvements in PFS (HR [95% CI] = 0.697 [0.595–0.818], 95% PI: 0.426–1.143; I^2^ = 66.0%, Tau^2^ = 0.0410) and OS (HR [95% CI] = 0.895 [0.809–0.990], 95% PI: 0.690–1.112; I^2^ = 19.2%, Tau^2^ = 0.0064), along with an increase in the ORR (RR [95% CI] = 1.444 [1.191–1.752], 95% PI: 0.874–2.389; I^2^ = 50.8%, Tau^2^ = 0.0325). Yet, VEGFR inhibitor combination therapy also increased the incidence of any grade AEs (RR [95% CI] = 1.023 [1.007–1.039], 95% PI: 0.965–1.075; I^2^ = 15.9%, Tau^2^ < 0.0001) and grade ≥3 AEs (RR [95% CI] = 1.259 [1.172–1.352], 95% PI: 0.720–2.667; I^2^ = 49.8%, Tau^2^ = 0.0291). Further analysis grouped by specific anti-angiogenic agents suggested that combination therapy with cediranib significantly improved PFS and ORR. A similar enhancement in ORR was observed with pazopanib combination therapy. The nintedanib combination therapy, while improving PFS, also escalated the risk of any grade AEs and grade ≥3 AEs (all *p* < 0.05). With regard to angiopoietin inhibitors, the combined therapeutic strategy significantly improved PFS (HR [95% CI] = 0.879 [0.798–0.968], 95% PI: 0.711–1.087; I^2^ = 0%, Tau^2^ = 0), but it also led to an increase in the occurrence of any grade AEs (RR [95% CI] = 1.015 [1.002–1.029], 95% PI: 0.991–1.031; I^2^ = 2.7%, Tau^2^ < 0.0001) and grade ≥3 AEs (RR [95% CI] = 1.068 [1.002–1.138], 95% PI: 0.966–1.178; I^2^ = 0%, Tau^2^ = 0). The identical results were also observed in the combination therapy with trebananib (Table 4; Supplementary Figure S10–S14).

### 3.7 Trial sequential analysis results

**FIGURE 4:**
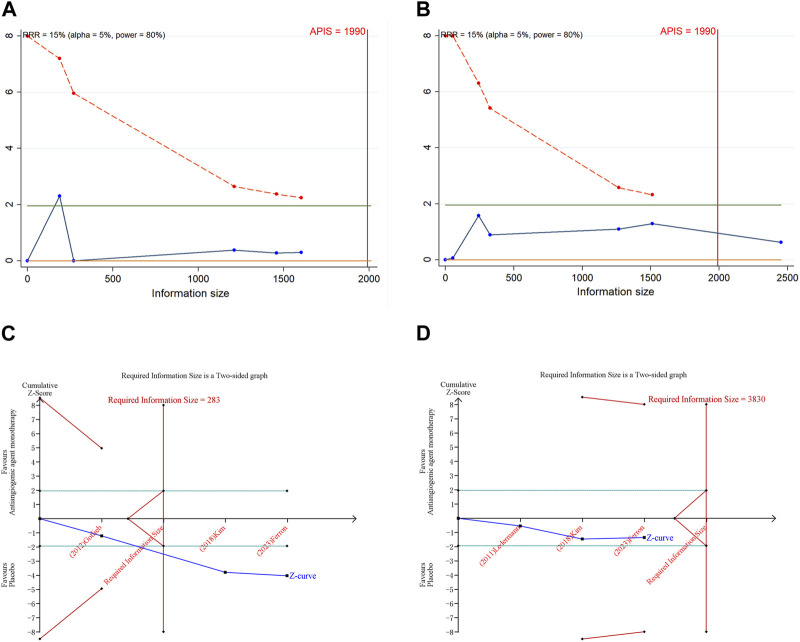
Trial sequential analysis of anti-angiogenic agent monotherapy for ovarian cancer. **(A)** Progression-free survival; **(B)** Overall survival; **(C)** Adverse events of any grade; **(D)** Grade ≥3 adverse events. Uppermost and lowermost red curves represent trial sequential monitoring boundary lines for benefit and harm, respectively. Inner red lines represent the futility boundary. Blue line represents evolution of cumulative Z-score. Horizontal green lines represent the conventional boundaries for statistical significance. Cumulative Z-curve crossing the trial sequential monitoring boundary or the RIS boundary provides firm evidence of effect.

**FIGURE 5:**
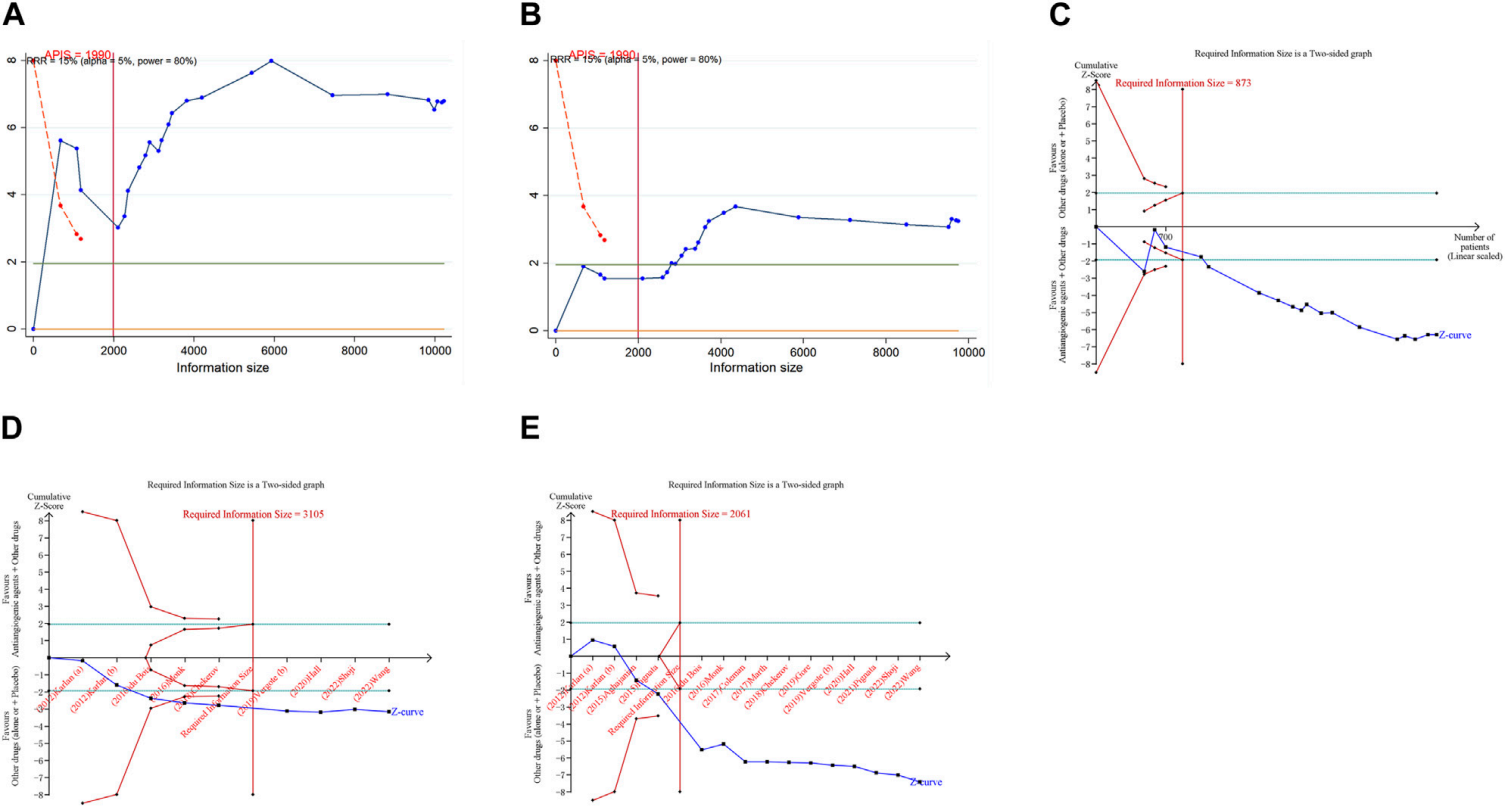
Trial sequential analysis of anti-angiogenic drug combination therapy for ovarian cancer. **(A)** Progression-free survival; **(B)** Overall survival; **(C)** Objective response rate; **(D)** Adverse events of any grade; **(E)** Grade ≥3 adverse events. Uppermost and lowermost red curves represent trial sequential monitoring boundary lines for benefit and harm, respectively. Inner red lines represent the futility boundary. Blue line represents evolution of cumulative Z-score. Horizontal green lines represent the conventional boundaries for statistical significance. Cumulative Z-curve crossing the trial sequential monitoring boundary or the RIS boundary provides firm evidence of effect.

In the execution of TSA for both PFS and OS, the analysis necessitated an APIS of 1,990. It was noted that in the monotherapy analysis with anti-angiogenic drugs, only the cumulative Z-curve for OS and any grade AEs breached the RIS threshold, albeit without breaching the trial sequential monitoring boundary. Theses results indicated the possibility of deriving a relatively solid conclusion. However, the cumulative Z-curve for PFS and grade ≥3 AEs in the same monotherapy analysis neither crossed the trial sequential monitoring boundary nor the RIS threshold, implying that the results are inconclusive and may include false positives (Figure 4). In the scenario of combination therapy with anti-angiogenic drugs, every cumulative Z-curve successfully crossed either the trial sequential monitoring boundary or the RIS threshold, suggesting that additional research is not necessary for a conclusive result (Figure 5).

### 3.8 Sensitivity analysis and publication bias

We performed sensitivity analyses and publication bias tests on the combined results that included more than 10 studies. The sensitivity analysis entailed the computation of pooled HRs or RRs along with their respective 95% CIs, excluding individual studies to ascertain if a single study significantly influenced the combined results. The sensitivity analysis demonstrated that the exclusion of any single study did not significantly impact the quantitative findings, which implies that the combined results from the anti-angiogenic drug combination therapy are both robust and dependable (Supplementary Figure S15). We also conducted a trim-and-fill analysis, yielding funnel plots with imputed studies for the outcomes of ORR, any grade AEs, and grade ≥3 AEs, indicating the potential for publication bias (Supplementary Figure S16). However, the trim-and-fill analysis correction for possible publication bias did not change the results for ORR, AEs of any grade, and grade ≥3 AEs, suggesting that the presence of publication bias did not significantly affect the final results.

## 4 Discussion

The progression of OC and the standard physiological processes of the ovary are both substantially reliant on angiogenesis. The growth and advancement of malignancies necessitate angiogenesis, as tumors cannot exceed 1–2 mm in size without adequate neovascularization. Consequently, anti-angiogenic drugs have been incorporated into OC treatment regimens. The VEGF pathway is the most extensively studied in the process of neovascularization. VEGF initiates the formation of new blood vessels, which is then sustained by platelet-derived growth factor, fibroblast growth factor, and angiopoietin-1 and -2 (Fernando et al., 2008; Timke et al., 2008; Ionescu et al., 2011). Overexpression of VEGF is associated with the tumor’s prognosis and stage (Nusrat et al., 2016). A number of angiogenesis inhibitors targeting this pathway, including bevacizumab, cediranib, sorafenib, pazopanib, aflibercept, nintedanib, trebananib, and sunitinib, are currently under investigation (Singh et al., 2020). This study conducted a meta-analysis of previous RCTs and concluded that compared to drug therapy without anti-angiogenic agents, combination therapy with anti-angiogenic drugs significantly improved PFS and OS, while also elevating the ORR. Further subgroup analysis revealed that combination therapy with VEGF or VEGFR inhibitors can bring benefits in terms of PFS and OS, as well as an improvement in ORR.

Bevacizumab is the main VEGF inhibitor of interest in the trials included in this study. This agent, a humanized monoclonal antibody targeting VEGF, received approval in 2014 as the treatment for platinum-resistant OC, to be used in conjunction with chemotherapy (Monk et al., 2016a). Our findings revealed that bevacizumab in combination with chemotherapy not only significantly improved PFS and OS but also increased ORR compared with chemotherapy alone or plus placebo in patients with OC. Bevacizumab achieves its therapeutic effect by preventing VEGF-A from engaging with VEGFR, resulting in the destruction of established vessels, interference with new vessel formation, and the reduction of intratumoral pressure (Reinthaller, 2016). Research indicated that inhibiting VEGF signaling not only diminishes tumor vascularization but also aids in the morphological and functional normalization of the remaining vessels (Mei et al., 2023). In addition, trebananib stands out as the sole angiopoietin inhibitor in our comprehensive analysis. This peptide, which obstructs the action of angiopoietin-1 and angiopoietin-2-key players in angiogenesis-acts by preventing ANGPT from interacting with its receptor, Tie2 (Mullen et al., 2019). Utilizing photoacoustic tomography, one study observed notable changes in tumor vascularization following trebananib treatment, including significant vessel regression and a decrease in vessel density. Notably, while trebananib therapy did not halt angiogenesis entirely, it encouraged the formation of more stable and less permeable residual vessels (Bohndiek et al., 2015). The TRINOVA-1 trial, assessing patients with recurrent OC less than 12 months after previous platinum-based therapy, allocated participants to either a combination of weekly paclitaxel and trebananib or weekly paclitaxel with placebo. The trebananib cohort experienced prolonged PFS (HR = 0.66, *p* < 0.001) (Monk et al., 2016b). Our analysis confirmed the benefit of trebananib combined with chemotherapy in improving PFS. Regrettably, this study did not demonstrate any significant improvement in OS and ORR when comparing trebananib plus chemotherapy to placebo plus chemotherapy.

Currently, VEGFR inhibitors attracting substantial clinical attention include cediranib, nintedanib, and pazopanib. Cediranib, an orally administered tyrosine kinase inhibitor (TKI), acts on VEGFR-1, -2, and -3, and c-kit. Preclinical OC models have demonstrated that cediranib therapy leads to a significant reduction in tumor vascular density and vessel regression (Ruscito et al., 2016). When combined with standard chemotherapy as a maintenance therapy, cediranib has demonstrated an extension in PFS and OS compared to chemotherapy alone (Mahner et al., 2015). When paired with the PARP inhibitor olaparib in patients with platinum-sensitive relapse OC, cediranib has exhibited a remarkable 80% response rate and an increase in PFS from 9 to 17.7 months (Liu et al., 2014). However, our combined analysis did not corroborate that cediranib combination therapy could enhance OS compared to treatments devoid of cediranib. Our research did affirm the benefit of cediranib combination therapy in extending PFS. Notably, the cediranib and olaparib combination therapy demonstrated a higher ORR compared to olaparib monotherapy in our study. Additional RCTs are needed to further probe the effectiveness of pairing anti-angiogenic drugs with PARP inhibitors in OC treatment. Nintedanib, a multi-targeted antiangiogenic agent available orally, has been shown through dynamic magnetic resonance imaging assessments to significantly reduce blood flow in approximately 55% of OC patients. It also fosters vascular normalization and tumor regression in pre-clinical models (Khalique et al., 2017). Nintedanib, when combined with carboplatin and paclitaxel, has been proven to improve PFS, although it has no effect on OS (Ray-Coquard et al., 2020). Pazopanib, an oral multi-target TKI, inhibits platelet-derived growth factor receptors (PDGFR) alpha/beta, VEGFR, c-Kit, and fibroblast growth factor receptor (FGFR)-1 and −3. In mouse orthotopic OC models, pazopanib treatment significantly curtailed tumor microvessel density and pericyte coverage (Merritt et al., 2010). While not yet approved for OC, numerous phase 2 and 3 clinical trials have explored the potential role of pazopanib in OC therapy (Davidson et al., 2014; du Bois et al., 2012; Plummer et al., 2013). Our research indicated that the combination of nintedanib and chemotherapy can improve PFS compared with chemotherapy alone or plus placebo. The combination of pazopanib and chemotherapy has been shown to provide higher ORR, which aligns with a previous meta-analysis (Zhang et al., 2023).

In addition to examining the impacts of various VEGF, VEGFR, and angiopoietin inhibitors on OC by classifying specific anti-angiogenic medications, our analytical approach distinguished itself from prior meta-analyses by performing subgroup analyses based on multiple OC subtypes (Wang et al., 2018; Guo et al., 2021). The results from our subgroup analysis suggested that compared to drug therapy without anti-angiogenic agents, combination therapy with anti-angiogenic drugs notably improved PFS, OS, and ORR in platinum-sensitive and recurrent OC patients. Traditionally, OC has been classified as “platinum sensitive” if relapse occurs 6 months or more after the final dose of platinum-based chemotherapy, and “platinum resistant” if relapse happens earlier (Ledermann et al., 2013). For platinum-resistant OC, our research also concluded that anti-angiogenic drug combination therapy yielded benefits in terms of PFS and OS, along with a higher ORR. Bevacizumab is the sole anti-VEGF treatment for platinum-sensitive and recurrent OC approved by the US Food and Drug Administration (FDA). Its FDA approved indication is for combination with carboplatin/gemcitabine or carboplatin/paclitaxel, followed by single-agent maintenance (Arend et al., 2020). Bevacizumab is also available in the United States as a second-line and third-line treatment for platinum-resistant OC and frontline therapy for stage III/IV disease (Arend et al., 2020). The majority of the participants in the RCTs included in our study were OC patients of various subtypes. Grouping the subdivided subtypes of OC into a single category in a general manner could lead to some degree of bias and confusion. Furthermore, the fifth Ovarian Cancer Consensus Conference of the Gynecologic Cancer InterGroup recommended that tumors should be defined by a multitude of factors, including surgical outcomes, mutation status, platinum sensitivity, histology, and response to non-platinum treatments. Consequently, more RCTs need to be incorporated to bolster future meta-analysis targeting a specific and clearly defined subtype of OC.

The results of our monotherapy analysis indicated that anti-angiogenic monotherapy did not provide substantial improvements in PFS and OS compared with placebo. This monotherapy, however, was associated with an elevated risk of any grade AEs. Despite the pooled analysis revealing a greater ORR with the use of anti-angiogenic monotherapy (Ferron et al., 2023), the inference made from a single trial could not be broadly applied. From a therapeutic efficacy standpoint, the combination of anti-angiogenic drugs with chemotherapy or PARP inhibitors seems to be a more effective alternative to monotherapy with anti-angiogenic drugs, as combination therapy brings benefits in terms of PFS, OS, and ORR. RCTs needs to be designed to directly compare the effectiveness of anti-angiogenic drug monotherapy and combination therapy to verify this hypothesis. Moreover, the increased incidence of AEs caused by combination therapy warrants attention. Our combined and subgroup analyses revealed that anti-angiogenic drug combination resulted in a higher incidence of grade ≥3 AEs. Additionally, the combination of VEGFR or angiopoietin inhibitor was linked to an increased risk of any grade AEs. These findings underscore the importance for vigilant monitoring and management of AEs during anti-angiogenic therapy to mitigate potential risks.

There are several limitations in our meta-analysis. First, the heterogeneity in PFS results could be attributed to the variations in the trial design, patient baseline characteristics, anti-angiogenic therapies utilized, chemotherapy protocols, OC stages, and duration of follow-up across the RCTs. The existence of considerable heterogeneity may compromise the dependability of pooled estimates. Second, it is noteworthy that despite the majority of the incorporated studies being featured in high-impact journals, certain inherent aspects like pharmaceutical industry sponsorship and an open-label design could potentially introduce elements of bias, such as publication bias, which might have an impact on the overall findings. Third, despite the participation of independent assessors and meticulous data extraction and quality assessment using the modified Jadad scale, subjective biases may still be present in the process of evaluating study quality and extracting data. Fourth, diversity in OC types across the original RCTs could make the subgroup analyses based on OC subtypes potentially biased and confusing. These subgroup analyses could potentially introduce the possibility of false positives and inflated type I error. Finally, TSA findings point out the need for future meta-analysis with larger sample sizes and more RCTs to validate the results related to PFS and grade ≥3 AEs in the context of anti-angiogenic drug monotherapy.

## 5 Conclusion

In summary, our meta-analysis of RCTs demonstrated that the combination of anti-angiogenic drugs with chemotherapy or PARP inhibitors significantly improved PFS, OS, and ORR in OC patients compared with chemotherapy or PARP inhibitors alone. Although the efficacy superiority of anti-angiogenic drug monotherapy over placebo has not been observed, the increased risk of AEs associated with anti-angiogenic drug monotherapy and combination therapy warrants attention. Clinicians should meticulously detect and manage AEs to mitigate the potential treatment-related risks while employing anti-angiogenic therapies.

## Data Availability

The original contributions presented in the study are included in the article/Supplementary Material, further inquiries can be directed to the corresponding author.
