# Supplementary material for: Efficacy and safety of anti-angiogenic drug monotherapy and combination therapy for ovarian cancer: a meta-analysis and trial sequential analysis of randomized controlled trials
Source: Front Pharmacol. 2024 May 27;15:1423891. doi: 10.3389/fphar.2024.1423891 (PMC11163095; doi:10.3389/fphar.2024.1423891)
Supplement: Supplementary file 1 [file Table1.DOCX]

| **PubMed 519** |
| --- |
| #1 (anti-angiogenic) OR (angiogenesis inhibitor) OR (antiangiogenetic) OR (anti-angiogenesis) OR (vascular endothelial growth factor) OR (VEGF) OR (VEGFR) OR (VEGF-R) OR (anti-VEGF) OR (Bevacizumab) OR (cediranib) OR (SU11248) OR (recentin) OR (Pazopanib) OR (AZD2171) OR (zaltrap) OR (GW786034) OR (AMG386) OR (Afibercept) OR (BIBF 1120) OR (Votrient) OR (axitinib) OR (vargatef) OR (AEE788) OR (Nintedanib) OR (BAY43-9006) OR (Avastin) OR (Sorafenib) OR (Imatinib) OR (STI571) OR (Sunitinib) OR (BAY 545-9085) OR (vandetanib) OR (Nexavar) OR (Trebananib) OR (Perifosine) OR (NSC724772) OR (AG-013736) |
| #2 (ovar*) AND (cancer* OR tumor* OR tumour* OR carcinoma* OR neoplasm* OR malignan*) |
| #3 (randomized controlled trial) OR (RCT) OR (controlled clinical trial) OR (random*) |
| #4 #1 AND #2 AND #3 |
| **Web of Science 1339** |
| #1 TS=((anti-angiogenic) OR (angiogenesis inhibitor) OR (antiangiogenetic) OR (anti-angiogenesis) OR (vascular endothelial growth factor) OR (VEGF) OR (VEGFR) OR (VEGF-R) OR (anti-VEGF) OR (Bevacizumab) OR (cediranib) OR (SU11248) OR (recentin) OR (Pazopanib) OR (AZD2171) OR (zaltrap) OR (GW786034) OR (AMG386) OR (Afibercept) OR (BIBF 1120) OR (Votrient) OR (axitinib) OR (vargatef) OR (AEE788) OR (Nintedanib) OR (BAY43-9006) OR (Avastin) OR (Sorafenib) OR (Imatinib) OR (STI571) OR (Sunitinib) OR (BAY 545-9085) OR (vandetanib) OR (Nexavar) OR (Trebananib) OR (Perifosine) OR (NSC724772) OR (AG-013736)) |
| #2 TS=((ovar*) AND (cancer* OR tumor* OR tumour* OR carcinoma* OR neoplasm* OR malignan*)) |
| #3 TS=((randomized controlled trial) OR (RCT) OR (controlled clinical trial) OR (random*)) |
| #4 #1 AND #2 AND #3 |
| **The Cochrane Library 1007** |
| #1 All Text=((anti-angiogenic) OR (angiogenesis inhibitor) OR (antiangiogenetic) OR (anti-angiogenesis) OR (vascular endothelial growth factor) OR (VEGF) OR (VEGFR) OR (VEGF-R) OR (anti-VEGF) OR (Bevacizumab) OR (cediranib) OR (SU11248) OR (recentin) OR (Pazopanib) OR (AZD2171) OR (zaltrap) OR (GW786034) OR (AMG386) OR (Afibercept) OR (BIBF 1120) OR (Votrient) OR (axitinib) OR (vargatef) OR (AEE788) OR (Nintedanib) OR (Avastin) OR (Sorafenib) OR (Imatinib) OR (STI571) OR (Sunitinib) OR (vandetanib) OR (Nexavar) OR (Trebananib) OR (Perifosine) OR (NSC724772) OR (AG-013736)) |
| #2 All Text=((ovar*) AND (cancer* OR tumor* OR tumour* OR carcinoma* OR neoplasm* OR malignan*)) |
| #3 All Text=((randomized controlled trial) OR (RCT) OR (controlled clinical trial) OR (random*)) |
| #4 #1 AND #2 AND #3 |
| **Embase 982** |
| #1 'anti angiogenic':ti,ab,kw OR 'angiogenesis inhibitor':ti,ab,kw OR antiangiogenetic:ti,ab,kw OR 'anti angiogenesis':ti,ab,kw OR 'vascular endothelial growth factor':ti,ab,kw OR vegf:ti,ab,kw OR vegfr:ti,ab,kw OR 'vegf r':ti,ab,kw OR 'anti vegf':ti,ab,kw OR bevacizumab:ti,ab,kw OR cediranib:ti,ab,kw OR su11248:ti,ab,kw OR recentin:ti,ab,kw OR pazopanib:ti,ab,kw OR azd2171:ti,ab,kw OR zaltrap:ti,ab,kw OR gw786034:ti,ab,kw OR amg386:ti,ab,kw OR afibercept:ti,ab,kw OR 'bibf 1120':ti,ab,kw OR votrient:ti,ab,kw OR axitinib:ti,ab,kw OR vargatef:ti,ab,kw OR aee788:ti,ab,kw OR nintedanib:ti,ab,kw OR 'bay43 9006':ti,ab,kw OR avastin:ti,ab,kw OR sorafenib:ti,ab,kw OR imatinib:ti,ab,kw OR sti571:ti,ab,kw OR sunitinib:ti,ab,kw OR 'bay 545-9085':ti,ab,kw OR vandetanib:ti,ab,kw OR nexavar:ti,ab,kw OR trebananib:ti,ab,kw OR perifosine:ti,ab,kw OR nsc724772:ti,ab,kw OR 'ag 013736':ti,ab,kw |
| #2 ovar*:ti,ab,kw AND (cancer*:ti,ab,kw OR tumor*:ti,ab,kw OR tumour*:ti,ab,kw OR carcinoma*:ti,ab,kw OR neoplasm*:ti,ab,kw OR malignan*:ti,ab,kw) |
| #3 'randomized controlled trial':ti,ab,kw OR rct:ti,ab,kw OR 'controlled clinical trial':ti,ab,kw OR random*:ti,ab,kw |
| #4 #1 AND #2 AND #3 |
