# Supplementary material for: Efficacy and safety of anti-angiogenic drug monotherapy and combination therapy for ovarian cancer: a meta-analysis and trial sequential analysis of randomized controlled trials
Source: Front Pharmacol. 2024 May 27;15:1423891. doi: 10.3389/fphar.2024.1423891 (PMC11163095; doi:10.3389/fphar.2024.1423891)
Supplement: Supplementary file 2 [file Table2.docx]

| Quality analysis of the included RCTs by modified Jadad scale. | | | | | | |
| --- | --- | --- | --- | --- | --- | --- |
| Study | Randomization | Randomization concealment | Double blind | Withdrawals and dropouts | Score | Study quality |
| Coleman 2017 | 2 | 2 | 0 | 1 | 5 | High |
| Pignata 2021 | 2 | 2 | 0 | 1 | 5 | High |
| Richardson 2018 | 2 | 2 | 2 | 1 | 7 | High |
| Monk 2016 | 2 | 2 | 2 | 1 | 7 | High |
| Aghajanian 2015 | 2 | 2 | 2 | 1 | 7 | High |
| Karlan 2012 | 2 | 2 | 2 | 1 | 7 | High |
| Nicum 2024 | 2 | 2 | 0 | 1 | 5 | High |
| Ledermann 2016 | 2 | 2 | 2 | 1 | 7 | High |
| Wang 2022 | 2 | 2 | 0 | 1 | 5 | High |
| Shoji 2022 | 2 | 2 | 0 | 1 | 5 | High |
| Gotlieb 2012 | 2 | 2 | 2 | 1 | 7 | High |
| Marth 2017 | 2 | 2 | 2 | 1 | 7 | High |
| Pignata 2015 | 2 | 2 | 0 | 1 | 5 | High |
| Chekerov 2018 | 2 | 2 | 2 | 1 | 7 | High |
| Liu 2019 | 2 | 2 | 0 | 1 | 5 | High |
| Pujade-Lauraine 2014 | 2 | 2 | 0 | 1 | 5 | High |
| Liu 2022 | 2 | 2 | 0 | 1 | 5 | High |
| Ledermann 2021 | 2 | 2 | 2 | 1 | 7 | High |
| Ferron 2023 | 1 | 1 | 2 | 1 | 5 | High |
| Burger 2011 | 2 | 2 | 2 | 1 | 7 | High |
| Aghajanian 2012 | 2 | 2 | 2 | 1 | 7 | High |
| Oza 2015 | 2 | 2 | 0 | 1 | 5 | High |
| du Bois 2016 | 2 | 2 | 2 | 1 | 7 | High |
| Ledermann 2011 | 2 | 2 | 2 | 1 | 7 | High |
| du Bois 2014 | 2 | 2 | 2 | 1 | 7 | High |
| Herzog 2013 | 1 | 1 | 1 | 0 | 3 | Low |
| Tewari 2019 | 2 | 2 | 2 | 1 | 7 | High |
| Vergote 2019a | 2 | 2 | 2 | 1 | 7 | High |
| Kim 2018 | 2 | 2 | 2 | 1 | 7 | High |
| Ray-Coquard 2020 | 2 | 2 | 2 | 1 | 7 | High |
| Vergote 2019b | 2 | 2 | 2 | 1 | 7 | High |
| Duska 2020 | 1 | 1 | 0 | 1 | 3 | Low |
| Roque 2022 | 2 | 2 | 0 | 1 | 5 | High |
| Hall 2020 | 1 | 1 | 0 | 1 | 3 | Low |
| Gore 2019 | 1 | 1 | 0 | 0 | 2 | Low |
