# Supplementary material for: Efficacy and safety of anti-angiogenic drug monotherapy and combination therapy for ovarian cancer: a meta-analysis and trial sequential analysis of randomized controlled trials
Source: Front Pharmacol. 2024 May 27;15:1423891. doi: 10.3389/fphar.2024.1423891 (PMC11163095; doi:10.3389/fphar.2024.1423891)
Supplement: Supplementary file 3 [file DataSheet1.docx]

**FIGURE S1** Subgroup analysis of progression-free survival after antiangiogenic agent monotherapy versus placebo for ovarian cancer. (A) Subgroup = Advanced ovarian cancer; (B) Subgroup = VEGFR inhibitors vs. Placebo; (C) Subgroup = Nintedanib vs. Placebo. (D) Subgroup = Pazopanib vs. Placebo.

**FIGURE S2** Subgroup analysis of overall survival after antiangiogenic agent monotherapy versus placebo for ovarian cancer. (A) Subgroup = Advanced ovarian cancer; (B) Subgroup = VEGFR inhibitors vs. Placebo; (C) Subgroup = Nintedanib vs. Placebo. (D) Subgroup = Pazopanib vs. Placebo.

**FIGURE S3** Subgroup analysis of any grade adverse events after antiangiogenic agent monotherapy versus placebo for ovarian cancer. (A) Subgroup = Advanced ovarian cancer; (B) Subgroup = VEGFR inhibitors vs. Placebo.

**FIGURE S4** Subgroup analysis of grade ≥ 3 adverse events after antiangiogenic agent monotherapy versus placebo for ovarian cancer. (A) Subgroup = Advanced ovarian cancer; (B) Subgroup = VEGFR inhibitors vs. Placebo. (C) Subgroup = Nintedanib vs. Placebo.

**FIGURE S5** Subgroup analysis of progression-free survival based on ovarian cancer (OC) subtypes after antiangiogenic agents plus other drugs versus other drugs (alone or plus placebo) therapy for OC. Subgroup = (A) platinum-sensitive and recurrent OC; (B) platinum-resistant OC; (C) newly diagnosed OC; (D) recurrent or persistent OC; (E) platinum-resistant or refractory OC; (F) advanced OC.

**FIGURE S6** Subgroup analysis of overall survival based on ovarian cancer (OC) subtypes after antiangiogenic agents plus other drugs versus other drugs (alone or plus placebo) therapy for OC. Subgroup = (A) platinum-sensitive and recurrent OC; (B) platinum-resistant OC; (C) newly diagnosed OC; (D) recurrent or persistent OC; (E) platinum-resistant or refractory OC; (F) advanced OC.

**FIGURE S7** Subgroup analysis of objective response rate based on ovarian cancer (OC) subtypes after antiangiogenic agents plus other drugs versus other drugs (alone or plus placebo) therapy for OC. Subgroup = (A) platinum-sensitive and recurrent OC; (B) platinum-resistant OC; (C) recurrent or persistent OC; (D) platinum-resistant or refractory OC; (E) advanced OC.

**FIGURE S8** Subgroup analysis of any grade adverse events based on ovarian cancer (OC) subtypes after antiangiogenic agents plus other drugs versus other drugs (alone or plus placebo) therapy for OC. Subgroup = (A) platinum-sensitive and recurrent OC; (B) platinum-resistant OC; (C) recurrent OC; (D) advanced OC.

**FIGURE S9** Subgroup analysis of grade ≥ 3 adverse events based on ovarian cancer (OC) subtypes after antiangiogenic agents plus other drugs versus other drugs (alone or plus placebo) therapy for OC. Subgroup = (A) platinum-sensitive and recurrent OC; (B) platinum-resistant OC; (C) recurrent OC; (D) advanced OC.

**FIGURE S10** Subgroup analysis of progression-free survival according to types of anti-angiogenic drugs after antiangiogenic agents plus other drugs versus other drugs (alone or plus placebo) therapy for ovarian cancer. Subgroup = (A) VEGF inhibitors + CT vs. CT (alone or + PL); (B) Bevacizumab + CT vs. CT (alone or + PL); (C) VEGFR inhibitors + Other drugs vs. Other drugs (alone or + PL); (D) Pazopanib + CT vs. CT (alone or + PL); (E) Cediranib + Other drugs vs. Other drugs (alone or + PL); (F) Nintedanib + CT vs. CT (alone or + PL); (G) Angiopoietin inhibitors + CT vs. PL + CT; (H) Trebananib + CT vs. PL + CT.

**FIGURE S11** Subgroup analysis of overall survival according to types of anti-angiogenic drugs after antiangiogenic agents plus other drugs versus other drugs (alone or plus placebo) therapy for ovarian cancer. Subgroup = (A) VEGF inhibitors + CT vs. CT (alone or + PL); (B) Bevacizumab + CT vs. CT (alone or + PL); (C) VEGFR inhibitors + Other drugs vs. Other drugs (alone or + PL); (D) Pazopanib + Paclitaxel vs. Paclitaxel (alone or + PL); (E) Cediranib + Other drugs vs. Other drugs (alone or + PL); (F) Nintedanib + CT vs. CT (alone or + PL); (G) Angiopoietin inhibitors + CT vs. PL + CT; (H) Trebananib + CT vs. PL + CT.

**FIGURE S12** Subgroup analysis of objective response rate according to types of anti-angiogenic drugs after antiangiogenic agents plus other drugs versus other drugs (alone or plus placebo) therapy for ovarian cancer. Subgroup = (A) VEGF inhibitors + CT vs. CT (alone or + PL); (B) Bevacizumab + CT vs. CT (alone or + PL); (C) VEGFR inhibitors + Other drugs vs. Other drugs (alone or + PL); (D) Pazopanib + CT vs. CT (alone or + PL); (E) Cediranib + Olaparib vs. Olaparib; (F) Angiopoietin inhibitors + CT vs. PL + CT; (G) Trebananib + CT vs. PL + CT.

**FIGURE S13** Subgroup analysis of any grade adverse events according to types of anti-angiogenic drugs after antiangiogenic agents plus other drugs versus other drugs (alone or plus placebo) therapy for ovarian cancer. Subgroup = (A) VEGF inhibitors + CT vs. CT (alone or + PL); (B) Bevacizumab + CT vs. CT (alone or + PL); (C) VEGFR inhibitors + CT vs. CT (alone or + PL); (D) Nintedanib + CT vs. CT (alone or + PL); (E) Angiopoietin inhibitors + CT vs. PL + CT; (F) Trebananib + CT vs. PL + CT.

**FIGURE S14** Subgroup analysis of grade ≥ 3 adverse events according to types of anti-angiogenic drugs after antiangiogenic agents plus other drugs versus other drugs (alone or plus placebo) therapy for ovarian cancer. Subgroup = (A) VEGF inhibitors + CT vs. CT (alone or + PL); (B) Bevacizumab + CT vs. CT (alone or + PL); (C) VEGFR inhibitors + CT vs. CT (alone or + PL); (D) Nintedanib + CT vs. CT (alone or + PL); (E) Angiopoietin inhibitors + CT vs. PL + CT; (F) Trebananib + CT vs. PL + CT.

**FIGURE S15** Sensitivity analysis of anti-angiogenic drug combination therapy for ovarian cancer. (A) Progression-free survival; (B) Overall survival; (C) Objective response rate; (D) Adverse events of any grade; (E) Grade ≥ 3 adverse events.

**FIGURE S16** Trim-and-fill analysis of anti-angiogenic drug combination therapy for ovarian cancer. (A) Progression-free survival; (B) Overall survival; (C) Objective response rate; (D) Adverse events of any grade; (E) Grade ≥ 3 adverse events.
